# Maturity and school outcomes in an inflexible system: evidence from Catalonia

**DOI:** 10.1007/s13209-019-0196-6

**Published:** 2019-07-08

**Authors:** Caterina Calsamiglia, Annalisa Loviglio

**Affiliations:** 1 ICREA and IPEG, Carrer Ramon Trias Fargas, 25-27 08005 Barcelona, Spain; 2 Universitat Autònoma de Barcelona and Barcelona GSE, Barcelona, Spain

**Keywords:** Human capital, Entry age, School cutoff date, I21, I28, H75

## Abstract

The existence of a rigid cutoff date which determines when children start primary school creates a large heterogeneity in students’ level of maturity within the classroom. We use rich administrative data of the universe of public schools in Catalonia to show that: (1) relatively younger children do significantly worse both in tests administered at the school level and at the regional level, and they experience greater retention. (2) These effects are homogeneous across SES and significant across the whole distribution of performance. (3) Younger children in our data exhibit higher dropout rates and choose the academic track in secondary school less often. (4) Younger children are more frequently diagnosed with learning disorders.

## Introduction

The fact that a unique school cutoff date determines when a child begins primary education induces large heterogeneity in the age at which children enter school. Older children will be substantially more mature than their younger peers, which may lead them to initially perform better. For instance, in Spain the school entry cutoff date is January 1, and children start primary education in September of the calendar year in which they turn 6 years old. Therefore, children born in January are about $20\%$ older than their peers born in December. In this paper, we use detailed administrative data for the Spanish region of Catalonia to provide evidence that age at enrollment in primary education affects students’ outcomes throughout their education.

Work by Heckman and co-authors shows that early child development is complementary to later learning—see Cunha et al. (2006) for a review. Bedard and Dhuey (2006) use international data to show that these initial maturity differences have long-lasting effects on student performance. Several papers look at the effects within a country: Fredriksson and Öckert (2014) for Sweden, Puhani and Weber (2007) for Germany, Schneeweis and Zweimüller (2014) for Austria, Black et al. (2011) for Norway, Crawford et al. (2010) for England, McEwan and Shapiro (2008) for Chile, Ponzo and Scoppa (2014) for Italy, and Elder and Lubotsky (2009) for the USA.

The international evidence agrees that relatively older students perform significantly better during primary education. The magnitude of the effect and the consequences for later outcomes seem to vary across countries. For instance, Black et al. (2011) show that in Norway the school starting age barely affects the results in a cognitive test at age 18 and does not matter for their prime-age earnings. Conversely, studies for countries with early tracking such as Germany (Puhani and Weber 2007) and Italy (Ponzo and Scoppa 2014) show that difference in performance is persistent and affects students’ allocation to academic or vocational education, which in turns will affect their labor market outcomes. This suggests that the longer run effect of age at enrollment depends upon the educational system in place.

The case of Spain, more specifically of Catalonia, deems particularly interesting for several reasons. First, children are not allowed to postpone or anticipate entrance to primary school, ensuring a clean identification. Second, grade repetition is common, especially during advanced grades of compulsory education. It is important to explore how age at entry and grade repetition interact. In fact, on the one hand, grade repetition might offset the effect of age at entry if it gives more time to less mature students for mastering the material. On the other hand, the empirical literature provides mixed evidence of its effectiveness in improving student performance (Fruehwirth et al. 2016) and suggests that when retention takes place in advanced grades, the probability of dropout substantially increases (Jacob and Lefgren 2009). Therefore, retention may widen the gap between younger and older students rather than closing it. Third, there is no tracking until the end of lower secondary education (tenth grade). In other countries, the effect of age at entry on performance or on the choice of dropping-out from school may be confounded with the different quality of the education offered by different tracks, especially if students’ maturity affects their selection into tracks. Fourth, after completing lower secondary education, students can choose whether to acquire further education, of either academic or vocational type. Only the former gives direct access to University; therefore, if age at entry affects students’ choice of enrolling in academic upper secondary education, it is very likely that it will affect their future educational and labor market outcomes.

While some previous cross-country studies include Spain among the countries under analysis, they can only analyze the effect of maturity on standardized assessments administered to a subsample of students who are in education at a given point in time.^1^ In this paper, we use administrative data of the universe of public schools in Catalonia for children enrolled in grades from 1 to 12 in the academic years 2009/2010–2013/2014. We study the effect of age at enrollment on several outcomes over time, including performance, retention, educational choices of whether to complete lower secondary education and whether to undertake upper secondary education, and diagnosis of learning disabilities.^2^

We find that relatively younger children are retained more often and perform worse both in tests administered at the school level and at the regional level. Particularly stark are the results on retention: while a small fraction of students are retained at the beginning of primary education (about 3% during the first two grades), those born at the end of the year are two times more likely to be retained than the average child. At age 14, they are almost 11 percentage points more likely to be in a lower school grade than their peers born at the beginning of the year. The negative effect of being younger on performance is decreasing as children get older, although significant throughout. In particular, the gap between younger and older children is almost 0.6 standard deviations when they are in second grade and still 0.2 standard deviations in middle school. The effect is significant and sizeable for all performance levels, as confirmed by quantile regressions. Grade repetition does not close the gap between younger and older students.

Contrary to what others have found for other countries, younger children in Catalonia drop out from education more often.^3^ A child born at the end of December is 2 p.p. more likely to leave lower secondary education without obtaining a diploma than a similar child born at the beginning of January. Moreover, younger students are less likely to enroll in academic upper secondary education. About 50% of students overall enroll in the academic track, but the probability of choosing it is 5 p.p lower for the youngest.

Finally, younger children are more frequently diagnosed with learning disabilities or attention-deficit disorders (e.g., ADD, ADHCD). The probability of being diagnosed for a child born at the end of the year is 50% higher than for an otherwise-identical child born at the beginning of the year.

In the literature, there is also evidence that the gap in achievement due to the age at enrollment may be larger for children coming from families with high socioeconomics (SES). In particular, Elder and Lubotsky (2009) show that in the USA, the impact of maturity is larger for high SES than for low SES. They argue that preschool experience changes with SES and hence the impact of spending one more year at home or in preschool is particularly benefiting for children coming from high SES. On the other hand, high SES could also help fill the gap during school years, by providing support and additional training to the relatively younger who are facing greater challenges. We find that in Catalonia the effect of maturity on performance is quite homogeneous by SES. This may be because there pre-primary education is guaranteed and free, and 97% of children attend preschool. This means that in the extra year before primary education older children from all family background spend a large fraction of their time in a relatively similar environment. Hence, there may be less heterogeneity due to the experience acquired in that additional year.

The rest of this paper is organized as follows. Section 2 describes the Catalan educational system and enrollment rules. Section 3 describes the data and discusses descriptive statistics. Section 4 outlines the empirical strategy. Section 5 presents the main results. Section 6 discusses robustness checks. Section 7 discusses heterogeneity analyses. Finally, Sect. 8 concludes.

## Catalan educational system and enrollment rules

Primary education (*Educació primaria*, corresponding to ISCED level 10) is the first stage of compulsory education in Catalonia; normally, a child begins primary school in September of the calendar year in which he or she turns 6 years old. In other words, the cutoff date is January 1. For instance, both a child born on January 1, 2003 and a child born on December 31, 2003 start primary school in September 2009, while someone born on January 1, 2004 starts 1 year later, in September 2010. This enrollment rule is quite sharp, and exceptions are extremely rare. Until 2008, they required the approval of the regional ministry of education.^4^ A reform of compulsory education approved in September 2008 introduced a slightly more flexible transition from preschool to primary school. The general rule is the same, but exceptions are managed by the school, together with the family instead of the department of Education.^5^ However, as shown in the next section, also in more recent years the overwhelming majority of children comply with the rule: more than 99% of them enroll in primary school at 6 years old.

Before primary education, children can attend preschool (*Educació infantil*) for 3 years (from September of the year in which they turn 3). Attendance is not compulsory, but in practice almost all families enroll their children.^6^ Normally, primary education takes 6 years, followed by 4 years of lower secondary education (*Educació secondaria obligatòria*, corresponding to ISCED level 24). Students are legally required to stay in school until they turn 16 or until they graduate. After successfully completing lower secondary education, students can enroll in upper secondary education for two more years. Upper secondary education is either academic (*Batxillerat*, corresponding to ISCED level 34) or vocational (*Grau Mitjà*, corresponding to ISCED level 35). Only students who complete academic upper secondary education can aim at enrolling in a 4-year bachelor degree in a University.

The rates of completion of secondary education and enrollment in further education are low in Catalonia (and more in general in Spain), in comparison with other European countries. According to Eurostat in years 2011–2013, about 25% of the population aged 18 to 24 in Catalonia attained at most lower secondary education and have not been involved in further education or training.^7^

Students whose performance is not sufficient can be retained and spend one more year in the same grade. By law, children can be retained at most once during primary education and at most twice during lower secondary education.^8^ Exceptions are allowed only for children with special educational needs and are extremely rare in practice.^9^ However, as shown in Sect. 3.2, retention is relatively infrequent during primary education and more common during lower secondary education.

## Data

Our analysis focuses on students enrolled in public schools in Catalonia from school year 2009/2010 to school year 2013/2014. Primary education and lower secondary education is typically provided by different schools. We call “primary schools” the providers of the former and “middle schools” the providers of the latter. Each year more than 50.000 children enroll for the first time in the first grade of a public primary school of the region. They are about 65% of the total number of students who enter primary education in that year.^10^

### Data sources and sample selection

We exploit data from different sources that provide us with detailed information on enrollment, school progression, academic outcomes and socio-demographic characteristics of Catalan students. The Institut Català d’Estadistica (IDESCAT) anonymized the data sources and provided us with student identifiers to merge them.

The *Departament d’Ensenyament* (regional ministry of education in Catalonia) provided enrollment records for the schools in the region, from preschool to high school. The IT infrastructure that supports the automatic collection of data has been progressively introduced since the school year 2009/2010. By year 2010/2011, almost all schools have already adopted it, while we have data for about 60% of them in 2009/2010.^11^ For all students in the public system, we observe the school and the class in which she is enrolled, the day of birth, the gender, the nationality, and whether she has special educational needs. Moreover, data include evaluations that the students receive at the end of grades 2, 4, and 6 (primary education), and at the end of each grade from 7 to 10 (secondary education), for each subject that they have undertaken. These evaluations are assigned by teachers taking into account the progression of the child and her performance in several tests administered during the cycle. For students enrolled in grade 10, i.e., the last grade of middle school, we can observe whether they graduated at the end of the year.^12^ Data also include some enrollment information and date of birth of students attending a private school, but neither other demographics nor their evaluations. Therefore, this paper focuses on students enrolled in public schools. “Appendix B” replicates some of the analyses for students enrolled in private schools.

The *Consell d’Avaluació de Catalunya* (public agency in charge of evaluating the educational system) provided us with the results of standardized tests taken by all the students in the region attending the last year of primary school (grade 6) and the last year of middle school (grade 10). Such tests are administered during spring since 2009/2010 for primary school and since 2011/2013 for middle school. They assess basic competences in Maths, Catalan, Spanish and English and have a purely statistical purpose: they do not affect the students’ final evaluations or progress to the next grades. We will refer to the results in these examinations, whose grading is blind, as *external evaluations*, in contrast to the final evaluations given by the school, that we will call *internal evaluations*.

Finally, we collect information on the student’s family background, more specifically on parental education from the Census (2002) and local register data (*Padró*). When the information could be retrieved from both sources, we imputed the highest level of education, presumably the most up-to-date information. We could not retrieve information about their parents for 4.5% of children, and those students are excluded from the analyses.

### Variables used and descriptive statistics

#### School and student characteristics

Table 1 describes the public education system in Catalonia in the academic year 2013/2014, the last in our sample. There are 1556 primary schools and 538 middle schools. On average, primary schools have 1.6 classes per grades, while middle schools have 3.3 classes per grade.^13^

About half of the students are female, 14.3% of students in primary education do not have Spanish citizenship (17.5% in lower secondary education).^14^ We define three categories of parental education: low, average, and high. Parents’ education is low if both parents have completed at most lower secondary education. It is high if one parent has tertiary education and the other has at least completed upper secondary education. It is average in the other cases.^15^ About 36.4% of students in primary education have low educated parents, while 27.2% have highly educated parents. Figures are 41.8% and 22.0%, respectively, for lower secondary education.

**Table 1:** Public schools in Catalonia

	Parents’ education			Female (%)	Immigrant (%)	Behind (%)	Special needs (%)	Schools	
	Low (%)	Avg (%)	High (%)					*N*	Classes
Primary S.	36.4	36.5	27.2	48.4	14.3	5.5	3.2	1556	1.6
Middle S.	41.8	36.2	22.0	48.6	17.5	22.6	3.2	538	3.3

Statistics computed for students enrolled in a public primary or middle school in Catalonia in the academic year 2013/2014

5.5% of primary school students are “behind”; namely, they are attending a lower grade than what normally expected for students of their age. The share is 8.7% among students who attend grade 6 (the last grade of primary school), while it rises to 16.2% in grade 7 (the first grade of middle school). Overall 22.6% of students in lower secondary education are behind. This suggests a sharp increase in grade repetition in middle school.

3.2% of students have special educational needs.^16^ This assignment is quite persistent: both in primary and secondary education, about 97% of students who are labeled as special needs in a given year have the same label the following period.

#### Evaluations

**Fig. 1:**
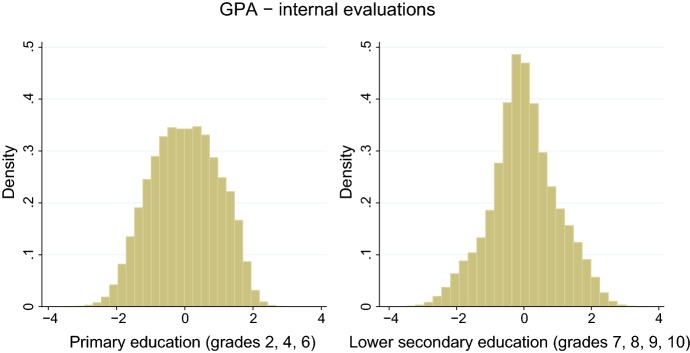
Distribution of evaluations. Distribution of GPA in primary schools (left panel) and middle schools (right panel)

The main measure of performance used in the analysis is the average of the evaluations in the four core subjects that all students undertake throughout compulsory education: Mathematics, Catalan, Spanish, and English. In lower secondary education, evaluations are assigned using a 0–10 scale, while in primary evaluations the marks “Fail,” “Pass,” “Good,” “Very good,” “Excellent” are used. We convert them into numeric values using the same scale of evaluations assigned in secondary education.^17^ Then, we compute *z*-score at the grade–year–school level. Standardizing the evaluations within school serves the purpose of improving comparability across schools. In fact, tests are designed and graded by the teachers of the school, and requirements to obtain a given score may vary substantially across schools. Figure 1 plots the distribution of GPA in primary education and secondary education.

Similarly, we compute the average score in Mathematics, Catalan, Spanish, and English for the region-wide test administered in grades 6 and 10. Then, we compute *z*-score at the grade–year level. Internal and external evaluations are positively correlated. The correlation is 0.77 in grade 6 and 0.62 in grade 10.

**Table 2:** Delayed or early enrollment in primary education

School year	Share who ...		*N* students
2009/2010	*Delays*	0.78%	
	*Anticipates*	0.064%	23,449
2010/2011	*Delays*	0.84%	
	*Anticipates*	0.045%	48,980
2011/2012	*Delays*	0.83%	
	*Anticipates*	0.036%	52,646
2012/2013	*Delays*	0.92%	
	*Anticipates*	0.056%	53,432
2013/2014	*Delays*	0.82%	
	*Anticipates*	0.049%	53,156
Total	*Delays*	0.84%	
	*Anticipates*	0.048%	231,663

For each year, the table shows the share of 6 years old who are still in preschool or are already in second grade. Last column shows the number of 6 years old in each year

#### School entry

Table 2 provides evidence that in all school years from 2009/2010 to 2013/2014, more than 99% of 6-year-old students are enrolled in the first grade of primary education. Moreover, there is not any evident trend that suggests an increasing attitude to postpone (or anticipate) entrance.^18^

We do not know the enrollment status at age 6 for children that were older when data collection started; therefore, we cannot infer the share of noncompliers for the previous school years. However, although we cannot assess the exact change due to the increased flexibility introduced by the decree issued in 2008, the overall effect of the change in law on enrollment behavior appears to be null or extremely small. Finally, it is worth noting that some immigrant students may have started their education abroad in a country with different enrollment rules, both for the cutoff date and for the flexibility of the system. In fact, the share of delayed enrollment is 0.71% among natives and 1.7% among immigrants. We will study whether the age effect is different for native and immigrant students in Sect. 7.

#### Age at entry and outcomes

**Fig. 2:**
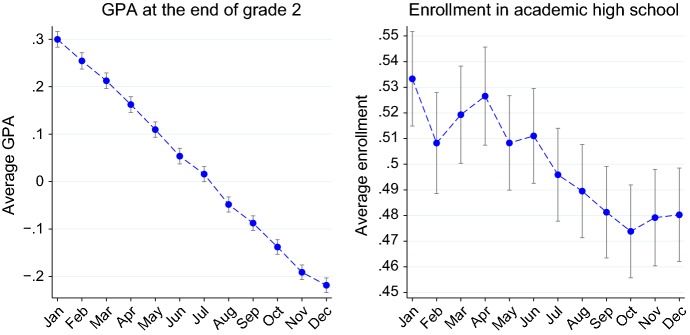
Mean outcomes by month of birth. The left panel plots the average GPA at the end of second grade for students born from 2002 to 2005. The right panel plots the share of enrollment in academic high school among students born in 1995

Figure 2 provides a first visual insight about the correlation between age at entry and educational outcomes in the short and longer run. The left panel plots the average GPA in second grade by month of birth: performances appear to be a linear decreasing function of the month of birth. On average, students born in January perform more than 0.5 standard deviations better than their peers born in December. The right panel plots the empirical probability of undertaking academic upper secondary education by month of birth. On average, younger children are less likely to enroll in academic high school than the older one (the difference between those born in January and those born in December is 5 percentage points), suggesting that the gap in maturity is persistent over time and has long-run consequences.

$A_i$, the main measure of age at entry used in this paper, is based on students’ day of birth. Days from 1 to 365 (or 366 in lap years) in the calendar year are mapped in the interval 0–1, so that January 1 corresponds to 1 and December 31 corresponds to 0. In fact, the largest difference in age at entry for compliers is 1 year. As explained in the previous subsection, almost all students comply with the rule of enrolling in primary school in the year in which they turn 6 years old. However, a very small fraction, typically less than 1%, postpone the entrance. Therefore, $A_i$, as inferred by student’s *i* date of birth, can be regarded as the “expected entrance age” in primary school, rather than the actual entrance age. We can observe the actual entrance only for students born from 2003 to 2005, who start primary education from 2009 to 2013, but not for those born in previous years.

## Empirical strategy

We study the effect of maturity at enrollment in primary school on children’ achievement throughout compulsory education and on their educational choices at the end of lower secondary education.

When analyzing the age effect on a continuous outcome, we rely on the following linear specification:

1 $$\begin{aligned} Y^t_i=\,&\alpha ^t A_i + X_i\gamma ^t +\epsilon ^t_i \end{aligned}$$

$Y^t_i$ is a measure of performance of student *i* in grade *t*, and we use either the GPA assigned by teachers or the score in the region-wide test (for grades 6 and 10). $X_i$ is a vector of time-invariant covariates realized before the child enrolls in school, including dummies for gender, immigrant status, parental education, and cohort (i.e., calendar year of birth). $\alpha ^t$ is consistently estimated under the assumption that $\mathrm {E}(\epsilon ^t_i|A_i, X_i)=0$. $A_i$, the age at enrollment in primary school, takes values in the interval 0–1; therefore, the estimated coefficient $\widehat{\alpha ^t}$ measures the difference in achievements between a student born on January 1 and a students born on December 31, everything else equal.

To be precise, $\alpha ^t$ captures both the effect of age at school entry and the effect of the age at which the children seat the test. While we cannot disentangle the two effects, we do not find this distinction to be particularly relevant in the current setting. In fact, internal evaluations are necessarily assigned at the end of the school year and have important consequences, both for retention decisions and for student self-assessment of ability.

When analyzing the age effect on a binary outcome $B_i$, we estimate a probit model

2 $$\begin{aligned} \Pr (B_i=1)&= \Pr (\alpha _B A_i + X_i\delta +\varepsilon _i\ge 0)= \Phi (\alpha _B A_i + X_i\delta ), \end{aligned}$$

where $\Phi$ is the CDF of the standard normal distribution. The binary outcomes we study are grade repetition, dropout, enrollment in high school, and special needs diagnosis. To ease the interpretation of coefficients, we compute average marginal effects and marginal effects for relevant subsamples of the population.

The crucial assumption to correctly identify the age effect is that the age at entry *A* is uncorrelated with unobserved characteristics that affect the outcomes of interest. This assumption would fail if, for instance, parents who care more about the school performance of their children target the first weeks of the year to give birth. In Sect. 4.2, we extensively discuss the plausibility of this assumption and we outline alternative specifications that we estimate as robustness checks.

If all students in our sample were observed when they started primary education, we could instrument the observed entrance age with the expected entrance age inferred from their date of birth. However, we observe most students for the first time in more advanced grades, and we only know their date of birth. Therefore, we rely on the “reduced form” approach described by Eqs. (1) and (2). “Appendix A” compares results of the reduced form model with a two-stage least square approach on the subset of students whose date of enrollment is directly observed. Given the extremely high rate of compliance, the two approaches are virtually identical. For the sake of simplicity, in the rest of this paper we will refer to $A_i$ as “age at enrollment” (or age at entry or entrance age), although we only observe the “expected” age.

### Conceptual framework

As Elder and Lubotsky (2009) point out, entrance age may have lasting effects on human capital for two reasons. First, all children in a class are exposed to the same educational methods and contents, but they may have different learning capabilities due to different levels of maturity. Second, even if at some point their current production functions are identical, their level of human capital may be different due to their past history, and therefore they may end up with different human capital in the following period. To clarify this point, let *A* be the age at enrollment and $Z^t$ other variables that contribute to human capital formation in grade *t* (for instance parental investment). Abstracting for now from grade repetition, let’s consider the following simple model of human capital accumulation:

3 $$\begin{aligned} H^{t}&=\beta H^{t-1} + \left( a^{t}A +Z^t b^{t}\right) \nonumber \\ H^1&=a^{1}A +Z^1 b^{1}, \end{aligned}$$

$H^{t}$, the human capital in period *t*, depends on past human capital and current inputs $Z^t$. If $a^{t}>0$, maturity has *direct* effect on human capital accumulation in grade *t*. In other words, we can say that children born in different months have access to different technology to produce human capital. If $\beta >0$, there is an *indirect effect* of *A* on human capital at time *t*, through its effect on the previous level of human capital. In particular, if $a^{t}=0$, *A* has only an *indirect* effect on $H^{t}$ through past human capital $H^{t-1}$. Replacing $H^{t-1}$ backward in (3), we obtain the following equivalent expression:

4 $$\begin{aligned} H^{t}&= \left( a^{t}+\sum _{k=1}^{t-1} \beta ^{t-k} a^k\right) A + \left( \sum _{k=1}^t \beta ^{t-k} Z^k b^k\right) , \end{aligned}$$

A test score $Y^{t}$ is a noisy measure of current level of human capital in grade *t*, i.e., $Y^{t}=H^{t}+\epsilon$. In practice, in the empirical analysis we rely on individual and family characteristics which are constant over time as proxy for inputs to human capital, bringing to the data the empirical specification in Eq. (1). The estimated coefficients capture the cumulative effects of the observed characteristics over time; in particular, $\alpha ^t=\left( a^{t}+\sum _{k=0}^{t-1} \beta ^{t-k} a^k\right)$ measures the *cumulative* effect of *A* on human capital in period *t*. We cannot disentangle the contemporaneous direct effect and the indirect effect accumulated over time, because $\beta$ is unknown. However, a comparison of the estimated coefficients across grades is informative of the prevailing effect: a decreasing sequence of $\widehat{\alpha ^t}$ would suggest that the main channel in the long run is the *indirect* effect and that the direct effect is only relevant in the initial years. In particular, if $\beta$ is sizeable, then $a^{t}$ must be almost insignificant in the future, only the past accumulation process being relevant in explaining the long-run gap.^19^

This simple illustration ignores grade repetition. Students who repeat a given grade may improve their stock of human capital at the end of that grade, and they are 1 year older when starting the following one. Grade repetition may counterbalance to some extent the effect of maturity, if younger students are more likely to be retained. We discuss this issue in Sect. 5.2.

### Identification

As explained in Sect. 5, the identification of the effect of age at enrollment *A* on student outcomes relies on the assumption that *A* is uncorrelated with unobserved characteristics that affect the outcomes. This assumption would not hold if, for instance, high-SES parents prefer to give birth in the first months of the year, or low-SES parents are more likely to deliver at the end of the year. In the analyses, we control for parent’s education, but this might not be enough if parents sort according to dimensions that are not perfectly captured by their education.

**Table 3:** Tests for continuity of the density of the day of birth

	All	Parents’ edu high	Parents’ edu avg	Parents’ edu low
McCrary (2008)	0.889	0.656	0.348	0.947
Calonico et al. (2014)	0.349	0.564	0.383	0.788
Frandsen (2017)	0.861	0.344	0.700	0.186

Tests are performed using the sample of students born from July 2002 to June 2005. Each entry in the table is a *p* value. We allow the McCrary (2008) test and the Calonico et al. (2014) test to select the optimal bandwidth independently. For the McCrary (2008) test, we set a bin size of 1 day, to account for the discrete nature of our running variable

In the first part of this subsection, we show that students are equally likely to be born at the beginning or at the end of the year, i.e., that there is no “manipulation” around the cutoff date of January 1. Moreover, we show that there is no difference in observable characteristics around the cutoff. In the second part of this subsection, we discuss issues related to birth seasonality. In fact, if mothers with certain characteristics are more likely to give birth in specific periods of the year, the estimated effects of age at entry may be confounded by birth seasonality even if there is no manipulation in a neighborhood of the cutoff date.

**Fig. 3:**
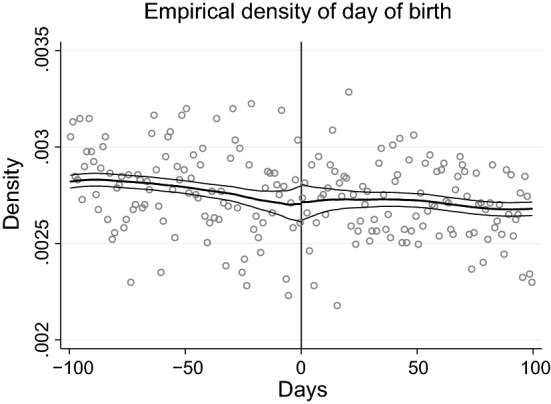
Distribution of births in the calendar year. Distribution of births around the cutoff date of January 1 (day 0). The figure plots the empirical density and confidence band estimated using the McCrary (2008) algorithm, using students born from July 2002 to June 2005

#### Distribution of births around the cutoff date

In the absence of manipulation, there should be no kinks in the density of the day of birth around the cutoff. We then formally test the no discontinuity hypothesis using three tests: the McCrary (2008) test, the Calonico et al. (2014) test, and the Frandsen (2017) test. To perform those tests, cohorts are defined as running from July 1 of a given year to June 30 of the following year. The running variable is the day of birth, with January 1 taking value 0, December 31 taking value − 1 and so on. We perform the tests on the sample of students born from July 2002 to June 2005, who are observed at ages 6, 7, and 8 in our data.^20^ Table 3 shows the *p* values of the three tests on the entire sample and on the three subgroups defined by parental education. All the *p* values are quite large, and the null hypothesis of no discontinuity is never rejected. Figure 3 plots the data and the estimated density and confidence interval using the McCrary (2008) algorithm, providing further visual evidence that there is no discontinuity around the cutoff date.

**Table 4:** Tests for balance of predetermined covariates

	(1)	(2)	(3)
Female	0.000	$-$ 0.000	$-$ 0.001
	(0.010)	(0.014)	(0.003)
Immigrant	0.003	$-$ 0.001	0.012
	(0.007)	( 0.011)	(0.002)**
Parents’ edu low	$-$ 0.006	0.003	0.019
	(0.010)	(0.015)	(0.003)**
Parents’ edu avg	0.015	$-$ 0.014	0.001
	(0.010)	(0.015)	(0.003)
Parents’ edu high	$-$ 0.002	$-$ 0.003	$-$ 0.016
	(0.009)	(0.012)	(0.003)**

**$p<0$.01. Tests are performed using the sample of students born from July 2002 to June 2005. For every line of the table, each entry reports the estimated difference of the covariate for students born before and after the cutoff date (January 1). The standard error of the estimate is reported in parenthesis. Columns (1) and (3) perform a *t* test for difference in mean; the sample for (1) includes only students born in the first 15 days of January and in the last 15 days of December, while all students are included in (3). Column (2) uses all the students in the sample and performs a local nonparametric RDD specification following Calonico et al. (2014). (We allow the algorithm to select the optimal bandwidth.)

Next, we provide evidence that the predetermined covariates (gender, immigrant status, parental education) are continuous around the cutoff date. First, we test whether the mean values are significantly different for students born around the cutoff date (more specifically in the last 15 days of December and in the first 15 days of January). As shown in column (1) of Table 4, we cannot reject the null hypothesis that there is no difference for any of the variables under analysis. Second, we use students born throughout the year to test whether any of the covariates is discontinuous around the cutoff date. More specifically, for each predetermined covariate $x_i$, we implement a regression discontinuity design using the following equation:

5 $$\begin{aligned} x_{i}&= \zeta D_i + \theta f(d_i) + \kappa D_i f(x_i) + c_i +\epsilon _i, \end{aligned}$$

where $D_i$ is a dummy that takes value 1 if the student is born between January 1 and June 30, $f(d_i)$ is a polynomial function of the day of birth, and $c_i$ is the cohort fixed effect. We estimate (5) using the nonparametric approach with a triangular kernel and a second order polynomial of the running variable, following the implementation proposed in Calonico et al. (2014).^21^ The parameter $\zeta$ captures difference in $x_i$ before and after the cutoff. We report estimate of $\zeta$ and standard errors in column (2) of Table 4. None of the covariates exhibits a statistically significant difference around the cutoff.

A further concern that may arise is that some parents may take in account the day of birth of their child when choosing between public and private schools. For instance, they may think that a private school would suit better the needs of a child born in December. This type of parents would be overrepresented in January and underrepresented in December in our sample of children in public schools. We use enrollment data to test whether the probability of being enrolled in a public primary school is different before and after the cutoff date. Both the simple difference in mean and the RDD approach cannot reject the null hypothesis that there is no difference. We cannot reject null hypotheses also when the sample for the tests is restricted to students with a given parental education.^22^

#### Birth seasonality and parental background

Results discussed so far support the hypothesis that children born around the cutoff date are not different at the beginning of their life. However, the distribution of births throughout the year may differ by parental background. In column (3) of Table 4, we test whether the mean values of the covariates are different for children born from July to December and those born from January to June. We find that the latter are 1.6 percentage points more likely to have highly educated parents and 1.2 percentage points more likely to be native Spanish.

**Fig. 4:**
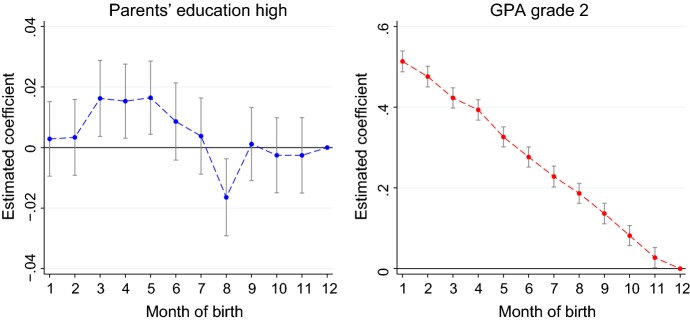
Correlation of month of birth with parents’ education and student’s performance. We regress a dummy for highly educated parents (left figure) or the GPA in grade 2 (right figure) on a vector of dummies for the month of birth, controlling for gender, immigrant status and cohort. The figures plot the estimated coefficients with their confidence interval

Equations (1) and (2) are linear in age at entry *A*. If students born in the first half of the year are more likely to have good educational outcomes because of their parental background, the estimated coefficients of age at entry *A* in Eqs. (1) and (2) may be upward biased. Figure 4 shows suggestive evidence that this should not be a concern. For the left panel of the figure, we regress a dummy for highly educated parents on a vector of dummies for the month of birth, controlling for gender, immigration status, and cohort. The graph plots the estimated coefficients with their confidence interval, showing that children born in March, April, and May are more likely to have highly educated parents. The opposite is true for those born in August, while there is no significant difference for the other months. If differences in educational outcomes between older and younger students are due to unobserved characteristics which are correlated with their parental background, we would expect to see a similar pattern when we regress their GPA on the dummies for the month of birth. Conversely, as shown in the right panel of the figure, the GPA is decreasing in the month of birth in a very linear way.

The analyses in Sect. 5 rely on the assumption that *A* has a linear effect on evaluations (Eq. 1) and on the latent variables of the binary outcomes studied with the probit model (Eq. 2). In Sect. 6, we discuss two alternative approaches to support our finding.

First, we replicate all the analysis using more flexible specifications. More specifically, we use dummies for the month of birth, or dummies for the week of birth.

Second, we replicate the analyses using only students born at the beginning of January and at the end of December, who have similar observable characteristics as shown in Table 4. We compare the estimated coefficients for an indicator for being born in January with the estimated coefficients from our baseline specifications. If the estimated effects are spuriously capturing unobserved differences between children born in different periods of the year, we would find much smaller effects when using the subsample of students born around the cutoff date.

It is important to remark that in this robustness check we are comparing outcomes of students born in January of a given calendar year with students born in December of the same year. We do not implement a regression discontinuity design, because it would require to compare students born in December of a given calendar year with students born in January of the following calendar year. However, those students belong to different cohorts: they begin their education 1 year apart, and they have different teachers, sit different examinations, and are potentially exposed to different educational reforms. These differences may bias the estimation in unpredictable ways.

## Main results

### Retention

We start our analysis by studying the effect of age at enrollment on probability of being retained during compulsory education. Results are shown in Table 5. Each column shows results of a probit specification that follows Eq. (2). We exploit two types of dependent variables. First, we use dummy variables that take value 1 if the student has been retained in any of the first two grades of primary education (first column) and in any of the first two grades of lower secondary education (second column). Studying the probability of retention in the following grades would be less informative, because, as explained in Sect. 2, students can be retained at most once during primary education and at most twice during lower secondary education. We exploit only cohorts for which we can observe both students who are behind and students that are progressing at the regular pace up to the second grade of primary or lower secondary education. Therefore, the sample for the analysis in the first column consists of students born from 2002 to 2005, and the sample for the second column consists of students born from 1997 to 1998.

**Table 5:** Grade repetition during compulsory education

	Repeat		Behind at		
	Grade 1 or 2	Grade 7 or 8	Age 8	Age 12	Age 14
Age at entry	− 0.637	− 0.163	− 0.698	− 0.597	− 0.382
	(0.023)**	(0.020)**	(0.019)**	(0.017)**	(0.012)**
	[− 0.043]	[− 0.038]	[− 0.059]	[− 0.088]	[− 0.109]
	<0.002>	<0.005>	<0.002>	<0.004>	<0.003>
Female	− 0.123	− 0.308	− 0.139	− 0.143	− 0.283
	(0.013)**	(0.013)**	(0.011)**	(0.009)**	(0.008)**
	[− 0.008]	[− 0.072]	[− 0.012]	[− 0.021]	[− 0.081]
	<0.001>	<0.003>	<0.001>	<0.002>	<0.002>
Immigrant	0.327	0.226	0.444	0.672	0.523
	(0.020)**	(0.019)**	(0.017)**	(0.027)**	(0.015)**
	[0.022]	[0.053]	[0.037]	[0.099]	[0.149]
	<0.001>	<0.004>	<0.002>	<0.006>	<0.004>
Parents’ edu avg	− 0.365	− 0.350	− 0.328	− 0.336	− 0.397
	(0.016)**	(0.015)**	(0.014)**	(0.018)**	(0.011)**
	[− 0.024]	[− 0.081]	[− 0.028]	[− 0.050]	[− 0.113]
	<0.001>	<0.004>	<0.001>	<0.003>	<0.003>
Parents’ edu high	− 0.704	− 0.854	− 0.596	− 0.661	− 0.899
	(0.023)**	(0.023)**	(0.020)**	(0.029)**	(0.018)**
	[− 0.047]	[− 0.199]	[− 0.050]	[− 0.098]	[− 0.256]
	<0.002>	<0.006>	<0.002>	<0.006>	<0.005>
*N*	163,910	72,079	205,242	188,075	178,871

**$p<0$.01. Cohort FE included. Standard errors in first and second columns (“Repeat...”) are clustered by school. Standard errors in the other three columns (“Behind at...”) are robust. Average marginal effects and their standard errors are reported in squared and angled brackets, respectively

Second, we assess the overall effect of maturity on the probability of being enrolled in a lower grade than the expected one given the year of birth. More specifically, we use dummies that takes value 1 if the student is behind at 8 years old (i.e., she is not yet in grade 3), if the student is behind at 12 (i.e., she did not start secondary education yet), and if she is behind at 14 (i.e., she is not yet in grade 9). Results are shown in the last three columns of Table 5. The chosen dummies are a close proxy for the event of having experienced retention at least once in the previous years, with the caveat that a student who started primary school late would be behind even if she was never retained during compulsory education.^23^ In the analysis, we exploit all observations belonging to students of a given age in years 2009–2013. For instance, we use students born from 2001 to 2005 to study the probability of not having reached grade 3 at 8 years old.^24^

On average, being 1 year younger increases probability of retention during the first two grades of primary school by 4.3 percentage points, a huge effect given that the average retention rate is about 3%.^25^ Children who were younger when starting primary school are also more likely to be retained several years later during lower secondary education. The average marginal effect of *A* on the probability of being retained in the first two grades of middle school is − 3.8 percentage points (16.5% of students are retained)

The remaining columns of Table 5 confirm that younger students are significantly less likely to progress regularly throughout compulsory education. More specifically, the average marginal effect of *A* on the probability of being behind at 8 years old is − 5.9 p.p. ( 4.3% of students are behind). Similarly, the average marginal effect on the probability of being behind at 12 (i.e., attending primary school rather than middle school) is 8.8 p.p. (9.2% of students are behind), and the one on the probability of being behind at 14 is 10.9 p.p. (24.8% of students are behind). Most of these sizeable effects should be attributed to increased probability of retention during compulsory education for younger students. In fact, as discussed in Sect. 3.2, overall less than 0.9% of students start primary education later, and the share is at most 2% in December.

### Evaluations

We study the effect of age at enrollment on academic performance, from second grade (in primary education) to tenth grade (in secondary education). Section 5.2.1 describes the subsamples used and the implementation of the analysis, Sect. 5.2.2 discusses the results, and Sect. 5.2.3 estimates quantile regressions to explore the effect of age at entry across the distribution of performance.

#### Sample selection

Performance is measured by internal evaluations in primary education (grades 2, 4, and 6) and lower secondary education (grades 7 to 10) and external evaluations at the end of each stage (grades 6 and 10). Given the limited time span of our sample, we cannot follow the same pool of students throughout compulsory education, but we rather use different cohorts of students to estimate the maturity effect $\alpha ^t$ for a given grade *t*.^26^

When analyzing internal and external evaluations in grade 10, a problem of selection arises: children aged 16 can drop out of school without completing lower secondary education. Given the retention rules discussed in the previous section, all children conclude at least grade 8, but they may drop out before concluding grade 10 or even grade 9. This means that the sample of students for which we can observe evaluations in the two last years of middle school is not fully representative of the initial population of children. This selection issue is discussed extensively in Sect. 5.3, where we analyze the effect of maturity on dropout. We include our results for grades 9 and 10 in the analysis discussed thereafter, in order to provide some illustrative evidence of the long-lasting effect of maturity on performance, but we acknowledge that we cannot exclude either positive or negative biases due to selection.

The subsample used for each regression includes only cohorts for which we can observe (at least) students with a regular progression or 1 year behind in primary education and up to 2 years behind in secondary education. Therefore, we use four cohorts of students to study performance in primary education and three cohorts for lower secondary education. For instance, when we study outcomes in second grade of primary school, we use students born from 2002 to 2005, while we do not include those who were born in 2001 (we would observe only students who are one or more year behind, but not those with a regular progression) or born in 2006 (we would observe only students in a regular progression, but not those retained the year before).^27^ This choice is aligned with the regulation of enrollment and progression in primary and secondary school: students can be retained at most once during the entire cycle of primary school and at most twice in secondary school. The region-wide test in grade 10 was introduced in the school year 2011/2012; therefore, we use only the cohort of students born in 1996 when external evaluations in lower secondary education are used as dependent variable.

Moreover, when a student undertakes the same grade twice, only the most recent evaluation is included in the analysis. Consider, for instance, a student who attended grade 2 in the year 2010 was retained and repeated the same grade in 2011, being finally promoted. The evaluation obtained in 2011 is included, while the other is discarded. This approach ensures that we use the evaluation that the student obtained when she is admitted to the following grade.^28^

Being retained affects performance both in the grade repeated twice and in the following periods. In fact, retained students have an additional year to learn the material covered in a given grade, and they are also 1 year older when they retake courses and examinations. For instance, about 85% of students improve their GPA when they retake a grade in primary school. Moreover, students who repeated grade *t* are older when they enroll in grade $t+1$ and, if retention is effective, they have a better stock of human capital than otherwise identical peers who did not repeat grade *t*. As shown in Sect. 5.1, younger students are more likely to be retained, and thus, retention may contribute to closing the gap due to the day of birth. In this case, $\alpha ^t$ in Eq. (1) is smaller than what would be found in an institutional setting without retention. Although such coefficients capture a combination of the effect of maturity on performance and on probability of being retained, results are still informative about the overall effect of the age at enrollment in primary school on the sequence of students’ evaluations in a system that allows grade repetition.

Precisely because our primary interest is to understand the longer term consequences of the initial maturity gap, in our baseline specification we use the last available outcome for retained children. That is, if a child has been retained in sixth grade, we analyze her evaluations the second time she takes the examinations in sixth grade. We also replicate the analysis using evaluations the first time students take the examinations. Comparison of the results gives us a sense of how much retention helps close the gap between older and younger students in a given grade.

**Table 6:** Evaluations in primary and lower secondary education

	Grade 2	Grade 4	Grade 6		Grade 7	Grade 8	Grade 9	Grade 10	
			(int.)	(ext.)				(int.)	(ext.)
*Panel A*									
Age at entry	0.574	0.407	0.329	0.351	0.220	0.175	0.153	0.137	0.219
	(0.008)**	(0.008)**	(0.009)**	(0.009)**	(0.010)**	(0.010)**	(0.010)**	(0.011)**	(0.021)**
Female	0.170	0.218	0.293	0.134	0.339	0.338	0.339	0.324	0.071
	(0.005)**	(0.005)**	(0.005)**	(0.006)**	(0.007)**	(0.007)**	(0.008)**	(0.008)**	(0.013)**
Immigrant	− 0.204	− 0.222	− 0.227	− 0.414	− 0.243	− 0.225	− 0.248	− 0.305	− 0.742
	(0.011)**	(0.011)**	(0.011)**	(0.014)**	(0.014)**	(0.014)**	(0.013)**	(0.013)**	(0.025)**
Parents’ edu avg	0.326	0.348	0.342	0.435	0.324	0.305	0.258	0.188	0.401
	(0.006)**	(0.007)**	(0.007)**	(0.010)**	(0.008)**	(0.009)**	(0.009)**	(0.008)**	(0.016)**
Parents’ edu high	0.621	0.670	0.679	0.837	0.681	0.661	0.591	0.492	0.854
	(0.008)**	(0.008)**	(0.009)**	(0.012)**	(0.012)**	(0.012)**	(0.011)**	(0.012)**	(0.020)**
$R^2$	0.12	0.12	0.12	0.16	0.13	0.12	0.11	0.09	0.21
*N*	160,438	148,472	136,281	117,752	106,707	102,960	96,170	86,025	24,465
*Panel B*									
Age at entry	0.583	0.412	0.334	0.350	0.220	0.170	0.154	0.144	0.226
	(0.009)**	(0.009)**	(0.009)**	(0.010)**	(0.010)**	(0.010)**	(0.011)**	(0.012)**	(0.023)**

**$p<0$.01. In panel A, cohort FE are included and standard errors clustered by school. In panel B, standard errors are robust

#### Results and alternative specifications

Each column of Table 6 (panel A) contains the estimated coefficients of a regression of a measure of performance on age *A*, individual characteristics, and cohort fixed effects, as in Eq. (1). For comparison, panel B reports results of a univariate regression of each dependent variable on *A*.

Table 6 provides compelling evidence that maturity is a very important determinant of students’ performance at the beginning of their school career: *ceteris paribus* being born at the beginning of January rather than at the end of December increases the GPA by 0.57 standard deviations. This effect is much larger than both the gender gap and the native-immigrant gap. It is just slightly smaller than the effect of having parents with tertiary education rather than with basic education.

The age effect is highly persistent over time, although decreasing in magnitude: it is 0.41 standard deviations in grade 4 and 0.33 s.d. in the last grade of primary education, while during lower secondary education being 1 year older is still associated with about 0.2 standard deviations increase in the GPA. Although the worst performing students, who (as we showed) are disproportionately born in the last months of the years, may have dropped out before reaching the fourth grade, age effect is still significant at 1% level in grade 10.

Columns (ext.) confirm that results are not driven by the particular evaluation procedure used in schools. The estimated effect is similar and if anything slightly larger using the test scores obtained by the students in their external evaluations.

A comparison of the estimated coefficients in panel A with those in panel B suggests that the age effect is orthogonal to socioeconomics. In fact, the estimates are almost unchanged when additional regressors are introduced.

In a cross-country analysis that exploits TIMSS and ECLS test scores, Bedard and Dhuey (2006) find that the effect of being born in January rather than in December ranges from 0.12 to 0.35 standard deviations in grade 4 and from 0.08 to 0.26 standard deviations in grade 8. Our results suggest that the age at entry effect for Catalonia is among the highest in the world.

We replicate the analysis in Table 6 separately by subjects, i.e., using as dependent variable the test score in Mathematics, Spanish, Catalan, or English. Results using the subjects rather than their GPA mimic the previous finding. Table 12 in Appendix C shows results for internal evaluations in grades 2, 6, and 7.^29^

We augment the specification for each grade with peer characteristics, including average age. We focus on primary education, because some middle schools may sort children across classes based on their performance.^30^ Results are shown in Table 13. Peers’ average age, the share of highly educated parents, and the share of females affect negatively the internal evaluations, while they have a positive effects on external evaluations in grade 6.^31^ To the extent that older students, students with high SES, and girls do better in school, these results are coherent with finding in Calsamiglia and Loviglio (2018), who show that being with better performing peers harms nonblind evaluations assigned by teachers. More importantly, for the purpose of this paper, coefficients of own regressors are virtually unaffected by the introduction of the new regressors, confirming the robustness of the baseline specification.

Finally, we replicate the analysis using evaluations the first time students take the examinations. Results, reported in Table 14, are quite similar to those in Table 6. Not surprisingly, the estimated $\widehat{\alpha ^t}$ are slightly larger in magnitude, but the increase is always smaller than 4% of the baseline estimate. For instance, the estimated age effect in grade 2 is 0.59 rather than 0.57 and in grade 8 it is 0.23 rather than 0.22.

The fact that the effect of maturity on school outcomes decreases over time supports the hypothesis that younger children create a lower stock of human capital in the earlier stage of their academic career, but later on they do not cumulate human capital at a lower rate for a given level of human capital from the previous period. However, the initial disadvantage is so large that the negative effects propagate over time and the gap is not closed at the end of lower secondary education.

A contrasting explanation is that the direct effect of maturity is still large in advanced grades, but the decreasing sequence of estimated coefficients is due to the higher number of younger kids who repeated a grade, partially closing the gap. While we cannot directly test the two alternative explanations, the latter appears unlikely for a number of reasons. First, we observe a clear decrease of $\widehat{\alpha ^t}$ also during primary school, when the retention rate is relatively low. Second, as just discussed, the improvement in evaluations due to retention in a given grade matters little for the estimates. Third, a large share of students perform well enough to be basically unaffected by the possibility of retention. In the next subsection, we perform quantile analysis to investigate the age effect at different points of the distribution of evaluations. The same evolution of the age effect over time is found for all the quantiles.

**Fig. 5:**
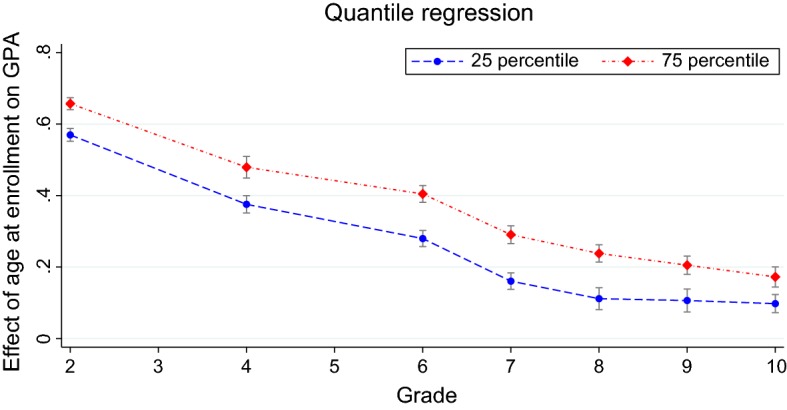
Effect of age at enrollment on GPA over time. The graph plots the results of quantile regressions of GPA (internal evaluations) on age at enrollment at the 0.25 and 0.75 quantile over school grades. Each blue dot is the estimated marginal effect for age at enrollment *A* at the 0.25 quantile in a given grade. Each red diamond is the estimated marginal effect for *A* at the 0.75 quantile. Gray bars are 95% confidence intervals (color figure online)

#### Quantile analysis

We perform quantile regressions to study how the effect of age at enrollment changes along the distribution of evaluations. We regress evaluations from grade 2 to grade 10 on age at enrollment *A* and the other covariates at various quantiles. Results are shown in Table 15. Figure 5 plots the estimated coefficients for the effect of age at enrollment on internal evaluations at the 25 percentile and 75 percentile. The evolution of the age effect over time is remarkably similar for the lower tail and the upper tail of the evaluations. As already observed for the linear regression model, the age effect is very large in grade 2, and it slowly decreases in the following grade, being still sizeable and significant at the end of lower secondary education. This pattern is similar at other quantiles, as shown in Table 15.

For each grade and type of evaluations, the age effect is sizeable and significant throughout the entire distribution. This is an important confirmation that we should be concerned about the negative effect of being younger at the time of enrollment for all children, not only for those who exhibit particular characteristics.

The age effect is somewhat increasing in the quantiles of internal evaluations, with a small drop at the very top of the distribution in primary school. Conversely, it is decreasing when external evaluations are used as outcome.^32^

Difference in the results for internal and external evaluations may be due to the fact that the former are more suitable to assess student performance at the top of the distribution, while the latter are more suitable to capture difference in performance at the bottom. External evaluations are meant to test basic knowledge, while internal evaluations may allow to discriminate better the middle–high part of the distribution rather than the lower tail. In fact, only one grade is available for insufficient performance in primary school; in middle school in principle numbers from 1 to 4 can be used, but 3 and 4 are much more frequent in practice.^33^

### Dropout and enrollment in academic high school

As discussed above, the age effect is persistent and still sizeable in middle school, but the trend is somehow decreasing over time. However, starting from the year in which they turn 16, students have to take decisions that will have long-lasting consequences on their labor market outcomes. First, if they turn 16 before graduation, they can drop out without concluding lower secondary education. Second, after completing lower secondary education, they can enroll in further education, of either academic or vocational type. It is of uttermost importance to understand whether the maturity effect is strong enough to affect their decisions: if the younger children make on average different choices than their elder counterparts, it becomes evident that the initial gap in age has long-term consequences. Thus, our goal in this section is to explore whether age at entry affects the probability of completing lower secondary education, and then the probability of enrolling in the academic track of high school, and the probability of choosing vocational education.

We can expect two opposed effects on probability of graduation. To the extent that low performers are more likely to drop out, we can expect age to have a positive effect on the probability of graduation. On the other hand, older students face a larger time period in which they can drop out; therefore, they may have higher incentives to leave school before graduation. Which effect prevails is an empirical question.

The limited time span covered by our data imposes some restrictions on the sample used for the current analysis. We focus on students born in 1995 and enrolled in a public middle school in 2009, and determine whether they graduate from a public school in the following years and whether they enroll in higher education.^34^

Although all children should attend school for at least some months before they turn 16 and can drop out, they may not be included in the records if schools transfer their data to the central system at the end rather than at the beginning of the academic year. Indeed, while in earlier years the probability of disappearing from the data in the following year is orthogonal with age, in the year in which they turn 14 children born at the beginning of the year are significantly more likely to disappear. Given this selection issue, to avoid to overestimate the age effect, we analyze outcomes of 14 years old rather than 15 years old.^35^

Seventy-three percent of 14-year-old students enrolled in a public school attend grade 9, while 24.7% are 1 year behind and 2.3% 2 years behind. We classify them as “graduate” if they complete lower secondary education after experiencing retention for at most one additional time. In other words, a student enrolled in grade 9 in 2009 should finish in 2011 at the latest, while a student enrolled in grade 8 should finish in 2012 at the latest, and one enrolled in grade 7 should finish in 2013.^36^ We adopt similar definitions for enrollment in academic or vocational upper secondary education.

Overall about 74% of students graduate, but students who are already behind at 14 years old are much more likely to drop out. In fact, only 42.6% of those who are in grade 8 complete lower secondary education, and for those who lag two grades behind the graduation rate is as low as 16.1%. 50% of 14 years old eventually enroll in academic upper secondary education, while 24% enroll in vocational training. Only 13% of students who are one or more grade behind at 14 enroll in the academic track.

We regress each of the binary variables for the events of graduation, enrollment in further academic education, and enrollment in vocational training, on age *A* and covariates using the probit model described in Eq. (2). Estimated coefficients and marginal effects are reported in Table 7.

**Table 7:** Probability of completing lower secondary education and undertaking further education

	Lower sec. edu.	Upper sec. edu.	
	Graduate	Academic	Vocational
Age at entry	0.068	0.162	− 0.108
	(0.028)*	(0.025)**	(0.025)**
	[0.020]	[0.056]	[− 0.033]
	<0.008>	<0.009>	<0.008>
Female	0.350	0.416	− 0.246
	(0.017)**	(0.016)**	(0.016)**
	[0.104]	[0.144]	[− 0.075]
	<0.005>	<0.005>	<0.005>
Immigrant	− 0.558	− 0.554	− 0.145
	(0.024)**	(0.024)**	(0.023)**
	[− 0.166]	[− 0.192]	[− 0.044]
	<0.007>	<0.008>	<0.007>
Parents’ edu avg	0.363	0.503	− 0.155
	(0.019)**	(0.017)**	(0.019)**
	[0.108]	[0.174]	[− 0.047]
	<0.006>	<0.006>	<0.006>
Parents’ edu high	0.747	1.110	− 0.547
	(0.029)**	(0.025)**	(0.024)**
	[0.223]	[0.385]	[− 0.166]
	<0.008>	<0.008>	<0.007>
*N*	33,624	33,624	33,624

*$p<0.05$; **$p<0.01$. Cohort FE included. Standard errors clustered by school. Average marginal effects and their standard errors are reported in squared and angled brackets, respectively

Age at entry has a significant effect on the probability of graduation. The average marginal effect of *A* is 2 percentage points. This finding suggests that as far as dropout is concerned, the “negative” effect of being younger (due to average worst academic performance and increased probability of retention) completely offsets the “positive” effect due to longer time of compulsory education. This is in contrast to previous studies that exploit US data. The seminal work by Angrist and Krueger (1991) shows that younger children are more likely to stay in school. In a recent paper, Cook and Kang (2016) find that, although older children obtain on average better evaluations before turning 16, they are then more likely to drop out and be engaged in criminal activities when adult. The peculiarity of the Spanish system, in which students who stay in school can achieve an official qualification a few months after they turn 16, may explain part of the difference in results: older children have more incentives to stay in school, and therefore, the “negative” effect of being younger is prevalent in our analysis.

Remaining columns of Table 7 show that maturity has a sizeable and significant effect on the probability of enrolling in further academic education: on average being 1 year older increases the probability of enrolling in further academic education by 5.6 percentage points and decreases the probability of enrolling in vocational training by 3.3 percentage points.

**Table 8:** Diagnosis of special needs

	Grades 1 and 2		Grades 7 and 8	
	Learning	Physical	Learning	Physical
Age at entry	− 0.256	− 0.024	− 0.217	0.039
	(0.025)**	(0.045)	(0.025)**	(0.057)
	[− 0.016]	[− 0.000]	[− 0.015]	[0.000]
	<0.002>	<0.001>	<0.002>	<0.001>
Female	− 0.334	− 0.053	− 0.289	− 0.011
	(0.015)**	(0.026)*	(0.016)**	(0.034)
	[− 0.021]	[− 0.001]	[− 0.020]	[− 0.000]
	<0.001>	<0.000>	<0.001>	<0.000>
Immigrant	0.006	0.024	0.069	0.036
	(0.028)	(0.039)	(0.022)**	(0.044)
	[0.000]	[0.000]	[0.005]	[0.000]
	<0.002>	<0.001>	<0.002>	<0.000>
Parents’ edu avg	− 0.213	− 0.028	− 0.361	− 0.095
	(0.021)**	(0.032)	(0.018)**	(0.042)*
	[− 0.013]	[− 0.000]	[− 0.025]	[− 0.001]
	<0.001>	<0.000>	<0.001>	<0.000>
Parents’ edu high	− 0.391	− 0.039	− 0.589	− 0.078
	(0.025)**	(0.035)	(0.027)**	(0.049)
	[− 0.024]	[− 0.001]	[− 0.041]	[− 0.001]
	<0.002>	<0.000>	<0.002>	<0.000>
*N*	149,142	149,142	115,204	115,204

*$p<0.05$; **$p<0.01$. Cohort FE included. Standard errors clustered by school. Average marginal effects and their standard errors are reported in squared and angled brackets, respectively

### Diagnosis of learning disorders

In public schools, children with special education needs may be granted additional support during compulsory education. As discussed in Sect. 3.2.1, each academic year less than 4% of students in our data are labeled as “special needs” children. While physical disabilities should be straightforward to identify, behavioral or learning disorders (such as attention-deficit or mild intellectual disability) are typically diagnosed while the student attends school, with teachers being instrumental in their detection and diagnose. After the condition is confirmed, the child is formally classified as needing special education arrangements.

Our concern here is that teachers’ perceptions may be partially clouded by children’s maturity: additional immaturity of a younger child may be confounded with a learning disability. Conversely, older children who would benefit from special support may be underdiagnosed. We provide evidence in support of this hypothesis, testing whether age at entry *A* affects the probability of being labeled with a “special needs” code related to learning disorder (development and behavioral disorders, mild intellectual disability, and other conditions whose detection may require some subjective judgment of the educator). As placebo test, we perform the same analysis using as dependent variable a dummy for physical disability (blindness, deafness, mobility issues, whose diagnosis should be relatively objective). Results are reported in Table 8. The first two columns refer to the first two grades of primary school, while the other refer to first two grades of middle school. We use students born in years 2003 to 2005 for primary education and students born from 1997 to 1999 for lower secondary education.

Being 1 year older reduces significantly the probability of being diagnosed with learning disorder in the first grades of primary school, and the effect is quite stable over time. In fact, the average marginal effect of age at entry is 1.6 percentage points in primary school and 1.5 percentage points in middle school. On the other hand, age at entry has no effect on the probability of being attributed physical disability.^37^

## Robustness checks

In the following subsections, we discuss the results of the robustness checks presented in Sect. 4.2. Moreover, in “Appendix A” we show that reduced form and instrumental variable approach deliver the same results. In “Appendix B”, we replicate some of the analyses discussed in Sect. 5 using students enrolled in private schools. Results are fully aligned with those for public schools, confirming that our findings hold for all students enrolled in the Catalan education system.

### Specifications flexible in the time of birth

We replicate all the analyses in Sect. 5 using dummies rather than one continuous variable for the age at entry. We use either dummies for the month of birth or dummies for the week of birth. For instance, we regress $Y^t_i$, the evaluations in grade *t*, on a vector of dummies for the month of birth and the usual covariates:

6 $$\begin{aligned} Y^t_i=\,&\sum _{m=1}^{11}\alpha _m^t M_{m,i} + X_i\gamma ^t +\epsilon ^t_i, \end{aligned}$$

where $M_{m,i}$ takes value 1 if student *i* is born in month *m*. December is used as reference category, and therefore, $\alpha _m^t$ is the expected difference in evaluations between a student born in month *m* and an otherwise-identical student born in December. We also run the same regression using dummies for the week of birth, and week 52 is used as reference category.

**Fig. 6:**
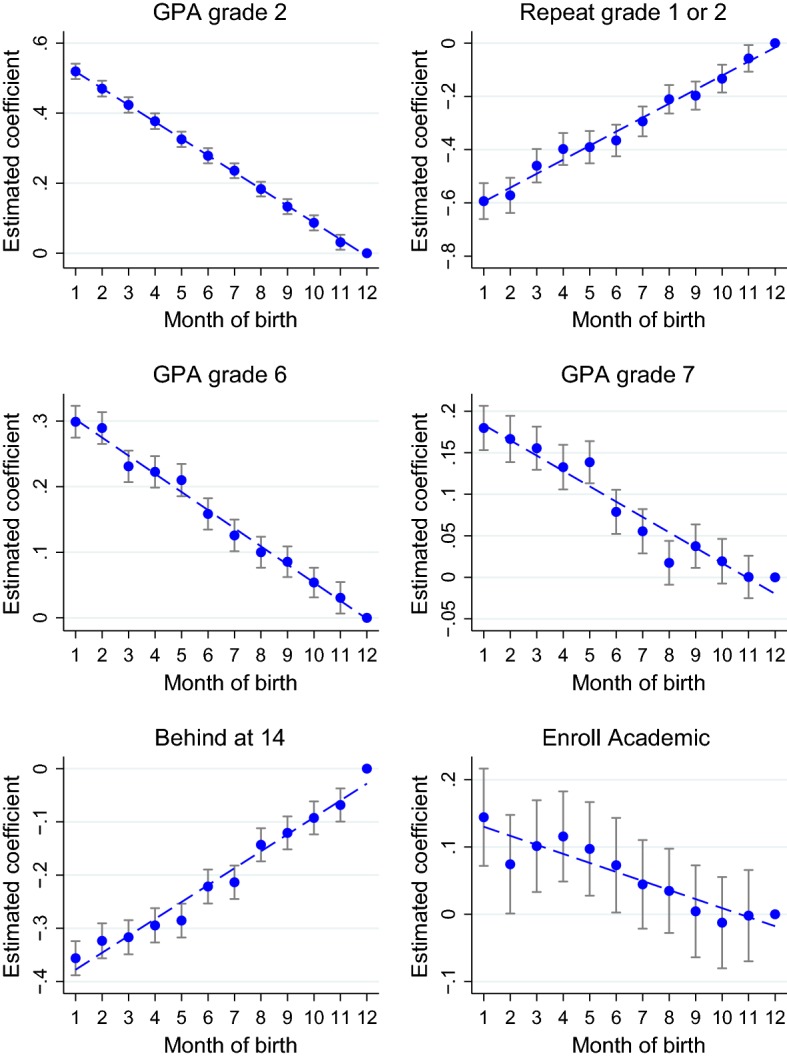
Estimated effect of month of birth on educational outcomes. The figures plot the estimated coefficients for the month of birth dummies and their confidence interval. December is the baseline category

**Fig. 7:**
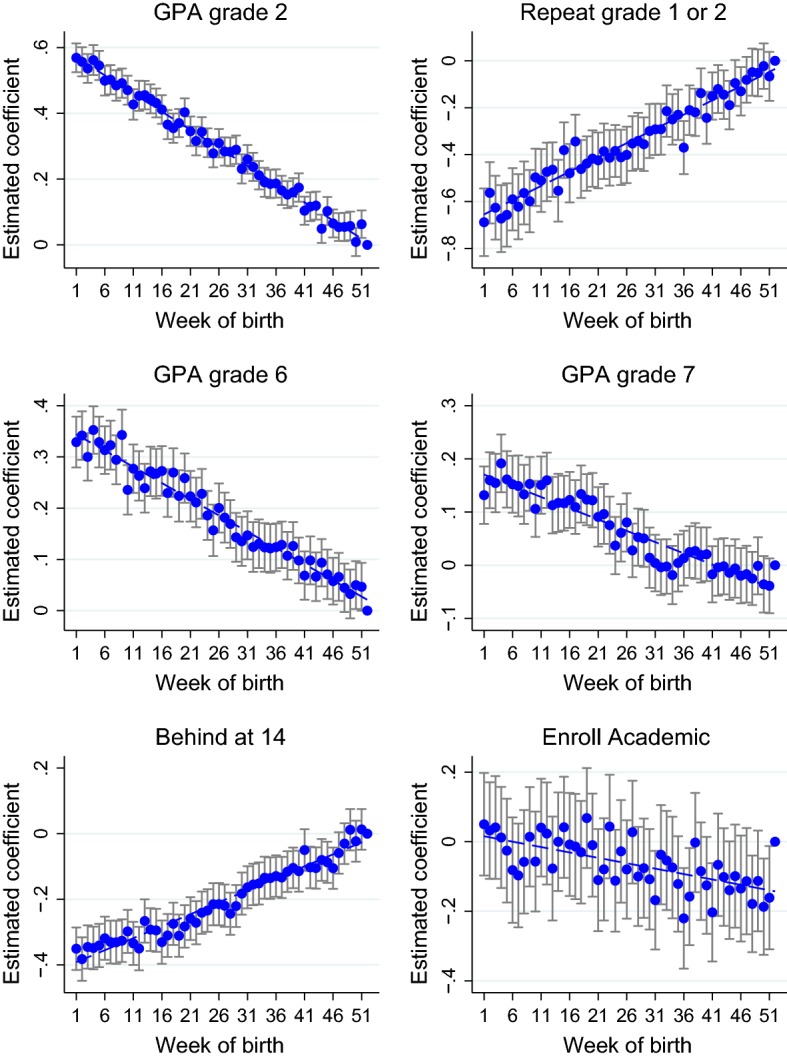
Estimated effect of week of birth on educational outcomes. The figures plot the estimated coefficients for week of birth dummies and their confidence interval. Week 52 is the baseline category

Figure 6 plots the estimated coefficients and confidence intervals of the month dummies for GPA in grades 2, 6 and 7, retention in the first two grades of primary school, being behind at 14 years old, enrollment in high school. Each graph displays also a linear fit of the estimated coefficients. The estimated coefficients for the January and November and for the other covariates are reported in columns (M) of Table 16. Figure 7 replicates Figure 6 using weekly dummies, and columns (W) of Table 16 show the coefficients.

Results using the month dummies confirm the findings discussed in Sect. 5. For each specification, the estimated effect of being born in January rather than in December is quite similar, only slightly smaller in magnitude, to the estimated effect of being 1 year older. Moreover, all outcomes are decreasing in the month of birth. The estimated effects on evaluations throughout compulsory education almost perfectly lie on their linear fit.^38^ The pattern is remarkably similar for the more demanding specifications that use the week dummies.^39^

### Analyses restricted to the subsample of students born around the cutoff

We replicate the analysis in Sect. 5 using only students born in the initial 15 days of January and in the last 15 days of December (16 for lap years). For instance, we regress $Y^t_i$, the evaluations in grade *t*, on a dummy $\text {old}_i$ and the usual covariates:

7 $$\begin{aligned} Y^t_i=\,&\alpha _\text {old}^t \text {old}_i + X_i\gamma ^t +\epsilon ^t_i, \end{aligned}$$

where $\text {old}_i$ takes value 1 if the student is born in January. $\alpha _\text {old}^t$ is the expected difference in evaluations in grade *T* between students born at the beginning and at the end of the year.

Results of the analyses on the much smaller sample of students born around the cutoff date are quite aligned with the results discussed in Sect. 5.^40^ More specifically, the estimated effects on evaluations are quite close to those found for the entire sample. For instance, $\alpha ^2=0.574$, while $\alpha _\text {old}^2=0.531$. The average marginal effects on the probability of being retained or being behind are the same or slightly larger in magnitude. The marginal effects on the probability of receiving a special needs diagnosis are also very similar to those found for the entire sample (e.g., 1.5 percentage points in primary school).

Estimated marginal effects on the probabilities of graduating and pursuing further studies are close enough. The average marginal effect on graduation is 3.5 percentage points (2 p.p. using the full sample), while the average marginal effect on enrollment in academic upper secondary education is 4.1 percentage points (5.6 p.p. for the full sample). The estimated marginal effect on enrollment in vocational education is − 1 percentage point but it is not significant (it is − 3.3 p.p. in the full sample). As explained in Sect. 5.3, those analyses are performed using only one cohort of students, those born in 1995; therefore, the sample size is relatively small. This may explain why the estimates are less precise than the ones discussed above.

## Heterogeneity analysis

We study whether the effect of age at entry is heterogeneous across subgroups of the population. We augment the specifications estimated in Sect. 5 with interaction terms between age at entry and parental education, gender, immigrant status.

**Table 9:** Marginal effects of age at entry by parents’ education

	Repeat grade 1 or 2		Behind at 14		Graduate		Enroll Academic	
	(1)	(2)	(1)	(2)	(1)	(2)	(1)	(2)
Parents’ edu. low	− 0.066	− 0.065	− 0.134	− 0.131	0.024	0.014	0.058	0.041
	(0.003)**	(0.004)**	(0.004)**	(0.006)**	(0.010)*	(0.012)	(0.009)**	(0.013)**
Parents’ edu. avg	− 0.035	− 0.038	− 0.106	− 0.113	0.020	0.044	0.061	0.096
	(0.002)**	(0.003)**	(0.003)**	(0.006)**	(0.008)*	(0.014)**	(0.009)**	(0.015)**
Parents’ edu. high	− 0.017	− 0.017	− 0.064	− 0.060	0.014	− 0.003	0.048	0.024
	(0.001)**	(0.002)**	(0.002)**	(0.006)**	(0.006)*	(0.015)	(0.007)**	(0.019)
All	− 0.043	− 0.043	− 0.109	− 0.109	0.020	0.021	0.056	0.057
	(0.002)**	(0.002)**	(0.003)**	(0.003)**	(0.008)*	(0.008)*	(0.009)**	(0.008)**

*$p<0.05$; **$p<0.01$. The table plots the average marginal effects of age at entry when parents’ education is set to be low, average, or high, and the overall average marginal effects (row “All”). Columns (1) use the baseline specifications estimated in Table 5 (for dependent variables “Repeat grade 1 or 2” and “Behind at 14”) and Table 7 (for dependent variables “Graduate” and “Enroll Academic”). Columns (2) use the specifications augmented with interaction terms in Table 18 (for dependent variables “Repeat grade 1 or 2” and “Behind at 14”) and Table 19 (for dependent variables “Graduate” and “Enroll Academic”)

### Retention and evaluations

As shown in Table 18, the effect of age on grade repetition or on the event of being behind does not exhibit significant difference by parents’ type. However, as reported in Table 9, the marginal effects of age at entry on retention probability are decreasing in parental education. For instance, being 1 year younger increases the probability of being behind at age 14 of about 13 percentage points for students with low educated parents, while the increases for students with highly educated parents are only 6 percentage points. This is not surprising, given the large effect that parental education has on the outcome: in the probit model, any change affects more those whose latent variable is closer to 0, i.e., whose probability is closer to 50%. For instance, 34% of students with low educated parents are behind at 14 years old and those shares are 18% and 8% for students with average and highly educated parents, respectively. Columns (1) and (2) of Table 9 confirm that the estimated marginal effects are almost identical for the baseline model and the model with interactions.^41^ In primary education, the estimated age effect on internal evaluations is slightly larger for students whose parents have an average level of education. As shown in Table 18, the difference is significant in grade 2 and 4, but it is small in size (e.g., it is 0.05 s.d. in grade 2, when the effect of being 1 year older is 0.54 for students with low educated parents). There are no differences between students with low- and high-educated parents. Conversely, in lower secondary education, the age effect is about 0.07 s.d. larger for students with high educated parents, while it is not significantly different for those with average education level. On the other hand, results using external evaluations in grade 6 show that age affects slightly less the performance of students with highly educated parents.^42^

The age effect is also similar for boys and girls. The effect on retention is slightly smaller in size for female students during primary education, while there is no significant difference in secondary education. The marginal effects are smaller for girls, who on average are less likely to experience retention. When evaluations are used as outcome, the estimated coefficients of the interaction term are positive but small in size, and only significant in lower secondary education.

Overall, the differences in the age effect on retention and evaluations by parental education or gender are small, if any. The age at entry has large impact on educational outcomes throughout compulsory education for both students with high and low socioeconomic status (SES), and for both boys and girls.

This finding contrasts with Elder and Lubotsky (2009), who find that in USA the effect is increasing in children’s SES.^43^ One possible explanation comes from the differences in preschool systems in Spain and USA. Preschool quality is quite heterogeneous in USA, and a substantial fraction of children do not attend preschool regularly. Parental contribution is therefore fundamental to determine the human capital level of the child at the beginning of formal education. Younger children start school having spent less time with their parents, and *ceteris paribus* the gap is larger among high SES, given that they have foregone more parental inputs when starting school. Conversely, in Spain almost every child is enrolled in preschool, for which there is free and universal access starting in the year in which the child turns 3. Even if the gap that we observe in primary school may be even larger in preschool, the quality of the time spent out of school before age 6 depends less on their SES.^44^

As shown in Table 18, the age effect on evaluations is significantly lower for immigrant students. The gap is larger in more advanced grades.^45^ A plausible explanation for this result is that many non-Spanish students are recent immigrants. They may have experienced a different educational system for the first part of their education, with different cutoff dates or simply more flexibility, and therefore they may be less affected by our measure of maturity at enrollment. Although this is a speculation we cannot directly test, it is supported by the evidence that the difference widens in more advanced grades, where recent immigrants have been exposed to relatively more education abroad than in Spain.

### Other outcomes

Table 19 replicates the analysis in Sects. 5.3 and 5.4, including the interaction terms in the specification. Results point to a larger effect of age at entry on dropout and enrollment in high school for students in the middle category of parental education. Conversely, there are no significant differences between students with low- and high-educated parents. For graduation, the estimated effect on the latent variable is almost double for students whose parents have average level of education. For this category, the estimated marginal effect on the probability of graduation is 4.4 percentage points, while it is 2 p.p. using the baseline model (Table 9). The coefficient of the interaction between age and the dummy for the middle category is also large and significant for the enrollment in further academic education. The estimated marginal effect is 9.6 percentage point, while it is 6 percentage point using the baseline specification. Similarly, there is a significant and sizable negative effect on enrollment in vocational training.

Completing lower secondary education and enrolling in high school are by a large extent a choice of the student and his or her family.^46^ Our findings are compatible with the hypothesis that family with average SES are more responsive to the level of skills of their children when taking educational decisions. For instance, this would be the case if high SES always push their children to acquire more academic education, even if they are not performing well in school, while low SES do not push them enough, even if they have the potential to succeed.

As discussed in the previous section, age at entry affects similarly performance of girls and boys. However, results in Table 19 suggest that age matters much more for boys than for girls for graduation and enrollment in academic upper secondary education. In fact, we cannot reject the null hypotheses that age at entry does not affect female graduation and enrollment.^47^ One possible reason for this results is that females are less responsive to their level of skills when choosing whether to drop out or to enroll in further education.

Results in the last two columns of Table 19 confirm that being younger increases the probability of being diagnosed with learning disabilities for all types of children. At the beginning of primary school, the estimated age effect is smaller for students with highly educated parents and it is larger for students whose parents have average educational level. However, they are the same in more advanced grades.

## Conclusions

Schools around the world are organized by cohorts; that is, children born in a given year are mixed in a classroom independently of their maturity or ability level. This induces an initial heterogeneity within the class that may affect individual development in the long run. This paper exploits an exogenous source of heterogeneity, i.e., the student day of birth, to show that an inflexible system that allows for no postponing of the entrance in primary school may lead to long-lasting consequences of the maturity effects.

We use administrative data from the universe of public schools in Catalonia to confirm the persistence of the disadvantage for younger children over time. The effect not only leads to worse performance over time, but to their choices as to whether to continue their education and what career to pursue to be significantly different. Although younger students are more likely to experience grade repetition, this does not seem to contribute much to close the gap, while it might negatively affect their probability of pursuing further education, given that they lag behind their peers when they turn 16.^48^ Moreover, age at entry negatively affects also children that are doing well in school, meaning that a high achiever born in December would have performed even better if she were born in January.

The fact that the effect is decreasing over time suggests that younger students initially create a lower stock of human capital and this negatively affects their human capital accumulation throughout compulsory education. Therefore, early interventions are likely to be the most effective. On the one hand, offering additional support or extra school time to less mature children may help reduce the initial heterogeneity, allowing for a better progression over time.

On the other hand, it appears desirable to have a flexible system in which readiness for school is assessed and school entrance can be postponed if deemed appropriate. However, interventions that increase flexibility may have unintended consequences if students who postpone the enrollment are allowed to drop out after spending less time in education. Thus, policymakers should also consider adjusting the rules for mandatory schooling to avoid those unintended interactions between postponed enrollment and the dropping-out decisions. In particular, in Spain education could be made mandatory until students complete lower secondary education. Alternatively, the current obligation of staying in school until a given age could be replaced with the requirement of undertaking at least a given number of years of basic education.

Finally, it is fundamental to involve teachers and school administrators and provide them with more information about the problem. In particular, we find that younger children are more likely to be labeled as “special needs” students. This suggests that it is important to raise the awareness of the school personnel, so that they routinely take in account their age at entry when assessing children during the initial grades of primary education.

## Appendix A: Two-stage least square regressions

As explained in Sect. 5, we do not observe the first enrollment in primary education for most of the students in our sample. Therefore, we infer the enrollment year from the date of birth. However, a subset of students (those born between 2003 and 2005) is observed both when they begin primary school and when they are evaluated at the end of their second year. We exploit this subset of children to compare two-stage least square estimates with our reduced-form approach.

**Table 10:** Evaluations and grade repetition: instrumental variable approach

	Evaluations		Repeat grade 1 or 2		First stage
	(RF)	(2SLS)	(RF)	(2SLS)	(2SLS)
Age at entry (expected)	0.570		− 0.040		0.988
	(0.009)**		(0.003)**		(0.001)**
Age at entry (observed)		0.577		− 0.041	
		(0.010)**		(0.003)**	
Female	0.161	0.163	− 0.011	− 0.011	− 0.003
	(0.006)**	(0.006)**	(0.001)**	(0.001)**	(0.000)**
Immigrant	− 0.162	− 0.165	0.027	0.027	0.005
	(0.013)**	(0.013)**	(0.003)**	(0.003)**	(0.001)**
Parents’ edu avg	0.330	0.331	− 0.028	− 0.028	− 0.002
	(0.008)**	(0.008)**	(0.002)**	(0.002)**	(0.001)**
Parents’ edu high	0.628	0.630	− 0.039	− 0.039	− 0.004
	(0.009)**	(0.009)**	(0.002)**	(0.002)**	(0.001)**
*N*	111,548	111,548	65,987	65,987	111,548

**$p<0.01$. In columns (2SLS), the observed enrollment age $O\!A_i$ is instrumented with the expected age $A_i$

Using the same notation of Sect. 5, and calling $O\!A_i$ the observed age at enrollment for individual *i*, the appropriate specification for evaluations $Y_i$ as linear function of $O\!A_i$ and other covariates $X_i$ is:

A-1 $$\begin{aligned} Y_i=\,&a O\!A_i + X_i b +u_i,\qquad cor(u_i,O\!A_i)\ne 0 \end{aligned}$$

A-2 $$\begin{aligned} O\!A_i =\,&\gamma A_i + X_i\delta + v_i,\qquad cor(v_i,A_i)= 0, \quad cor(u_i,A_i)= 0 \end{aligned}$$

The two-stage least-squares estimation estimates first $\widehat{\gamma }$ and $\widehat{\delta }$ in Eq. (A-2) and then replaces $O\!A_i$ in Eq. (A-1) with $\widehat{O\!A_i}=\widehat{\gamma }A_i +X_i\widehat{\delta }$. Given that $\widehat{A_i}$ is uncorrelated with the error $(u_i+v_i)$, this makes possible a consistent estimation of *a*.

The reduced-form approach consists in replacing age in Eq. (A-1) with the RHS of Eq. (A-2):

A-3 $$\begin{aligned} Y_i=\,&a\gamma A_i+X_i(b+a\delta ) + \alpha v_i+ u_i \end{aligned}$$

A-4 $$\begin{aligned} =\,&\alpha A_i+X_i\beta +\epsilon _i,\qquad cor(\epsilon _i,A_i)= 0 \end{aligned}$$

$\alpha =a\gamma$ can be consistently estimated, but in general it is different from *a* if $\gamma \ne 1$. In our case, however, $\gamma$ is really close to 1, because $O\!A_i=A_i$ for 99% of students. Therefore, we can expect $\widehat{\alpha }$ to deliver a good approximation of *a*, even if slightly smaller. The intuition is confirmed by results in Table 10. The first two columns of the table compare estimated coefficients for the regression of evaluations in grade 2 on age at entry and covariates. Column (RF) replicates the first column of Table 6 using the subsample of students born from 2003 to 2005. In column (2SLS), the observed age at enrollment $O\!A_i$ is instrumented with the expected age $A_i$.

The estimated effect of age at entry is almost the same using the two approaches: it is 0.57 with the reduced-form estimation and 0.58 with the two-stage using instrumental variable approach. The third and fourth columns compare results for grade repetition in the first two grades of primary education, using the subsample of students born in 2003 and 2004. Again, the estimated effect is almost the same (− 4 p.p. and − 4.1 p.p., respectively). To conclude, we can regard the estimations obtained by reduced form as a lower bound of the true effect.

**Table 11:** Evaluations and grade repetition: private schools

	Evaluations gr. 6		Evaluations gr. 10		Behind at 14		Enroll Academic	
	(1)	(2)	(1)	(2)	(1)	(2)	(1)	(2)
Age at entry	0.339	0.329	0.174	0.114	− 0.374	− 0.394	0.153	0.142
	(0.007)**	(0.011)**	(0.015)**	(0.020)**	(0.009)**	(0.017)**	(0.021)**	(0.039)**
					[− 0.099]	[− 0.085]	[0.053]	[0.043]
					<0.002>	<0.004>	<0.007>	<0.012>
Public school	− 0.240		− 0.352		0.184		− 0.389	
	(0.013)**		(0.019)**		(0.006)**		(0.024)**	
					[0.049]		[− 0.134]	
					<0.002>		<0.008>	
Parents’ edu avg	0.459	0.386	0.446	0.357	− 0.450	− 0.442	0.548	0.556
	(0.008)**	(0.015)**	(0.014)**	(0.021)**	(0.006)**	(0.011)**	(0.015)**	(0.030)**
					[− 0.119]	[− 0.095]	[0.189]	[0.169]
					<0.002>	<0.002>	<0.005>	<0.009>
Parents’ edu high	0.840	0.711	0.873	0.707	− 0.963	− 0.912	1.167	1.130
	(0.011)**	(0.019)**	(0.018)**	(0.024)**	(0.008)**	(0.012)**	(0.021)**	(0.037)**
					[− 0.255]	[− 0.196]	[0.402]	[0.344]
					<0.002>	<0.003>	<0.006>	<0.010>
*N*	190,371	73,287	42,816	18,137	292,500	112,421	48,959	15,490

**$p<0.01$. In columns (1), both students enrolled in public and private schools are included in the analysis. In columns (2), only students enrolled in private schools are included

## Appendix B: Private schools

Some of the analyses discussed in Sect. 5 can be replicated exploiting also data for students enrolled in private schools. Results are shown in Table 11. The regressions in columns (1) are performed using both students enrolled in public and private schools, while those in columns (2) are restricted to students in private schools.

Being 1 year older increases external evaluations in grade 6 of students enrolled in private schools by 0.33 standard deviations. The estimated coefficient is quite similar to the one found for public schools. In grade 10, the effect is significant and sizeable (0.11 standard deviations), but somewhat smaller than what found for public schools. However, it is important to recall that only students who do not drop out undertake evaluations in grade 10; therefore, some selection bias may affect the estimate.

The age at entry has a large effect also on the probability of being behind at 14 (the average marginal effect is − 8.5 p.p.) and enrolling in academic upper secondary education (the average marginal effect is 4.3 p.p.). Those figures are comparable with the finding for public schools. It is not surprising that they are slightly smaller because, as shown in column (1), students enrolled in private schools are less likely to be retained and more likely to undertake academic education.

## Appendix C: Additional tables

See Tables 12, 13, 14, 15, 16, 17, 18, and 19.

**Table 12:** Evaluations by subjects

	Mathematics			Catalan			Spanish			English		
	2	6	7	2	6	7	2	6	7	2	6	7
Age at entry	0.536	0.299	0.201	0.504	0.308	0.198	0.529	0.311	0.206	0.489	0.284	0.195
	(0.008)**	(0.009)**	(0.010)**	(0.008)**	(0.009)**	(0.010)**	(0.009)**	(0.009)**	(0.009)**	(0.008)**	(0.009)**	(0.009)**
Female	− 0.082	0.066	0.157	0.229	0.336	0.365	0.249	0.353	0.364	0.230	0.325	0.354
	(0.005)**	(0.006)**	(0.007)**	(0.005)**	(0.005)**	(0.007)**	(0.005)**	(0.005)**	(0.007)**	(0.005)**	(0.006)**	(0.007)**
Immigrant	− 0.158	− 0.174	− 0.201	− 0.232	− 0.257	− 0.243	− 0.275	− 0.265	− 0.262	− 0.091	− 0.151	− 0.191
	(0.011)**	(0.011)**	(0.013)**	(0.011)**	(0.011)**	(0.014)**	(0.011)**	(0.011)**	(0.013)**	(0.011)**	(0.011)**	(0.014)**
Parents’ edu avg	0.297	0.312	0.297	0.301	0.312	0.283	0.285	0.313	0.290	0.292	0.319	0.309
	(0.006)**	(0.007)**	(0.008)**	(0.006)**	(0.007)**	(0.008)**	(0.006)**	(0.007)**	(0.008)**	(0.006)**	(0.007)**	(0.008)**
Parents’ edu high	0.571	0.633	0.636	0.590	0.633	0.615	0.486	0.589	0.588	0.573	0.635	0.642
	(0.008)**	(0.009)**	(0.012)**	(0.008)**	(0.009)**	(0.012)**	(0.008)**	(0.009)**	(0.012)**	(0.008)**	(0.009)**	(0.011)**
$R^2$	0.09	0.09	0.09	0.11	0.12	0.12	0.10	0.12	0.12	0.10	0.11	0.12
*N*	162,048	137,300	108,389	161,910	137,059	108,349	161,874	137,162	108,153	160,782	137,023	106,938

**$p<0$.01. Cohort FE included. Standard errors clustered by school. For each column, the sample used is the same than in Table 6

**Table 13:** Evaluations in primary education: peer characteristics included as regressors

	Grade 2	Grade 4	Grade 6	
			(int.)	(ext.)
Age at entry	0.575	0.409	0.332	0.349
	(0.008)**	(0.008)**	(0.009)**	(0.009)**
Female	0.168	0.218	0.291	0.135
	(0.005)**	(0.005)**	(0.005)**	(0.006)**
Immigrant	− 0.308	− 0.322	− 0.328	− 0.333
	(0.010)**	(0.010)**	(0.010)**	(0.011)**
Parents’ edu avg	0.383	0.403	0.392	0.396
	(0.006)**	(0.006)**	(0.006)**	(0.009)**
Parents’ edu high	0.747	0.799	0.807	0.736
	(0.007)**	(0.007)**	(0.008)**	(0.009)**
Mean age at entry	− 0.523	− 0.335	− 0.270	0.183
	(0.025)**	(0.028)**	(0.030)**	(0.071)**
% females	− 0.158	− 0.205	− 0.235	0.141
	(0.014)**	(0.016)**	(0.018)**	(0.050)**
% immigrants	0.469	0.466	0.429	− 0.322
	(0.015)**	(0.014)**	(0.014)**	(0.048)**
% parents’ edu high	− 0.629	− 0.667	− 0.693	0.571
	(0.011)**	(0.011)**	(0.013)**	(0.042)**
$R^2$	0.15	0.15	0.15	0.17
*N*	160,438	148,472	136,281	117,752

**$p<0$.01. Cohort FE included. Standard errors clustered by school. Peer characteristics are computed at the class level. For each column, the sample used is the same than in Table 6

**Table 14:** Evaluations in primary and lower secondary education: using first-time evaluations for repeaters

	Grade 2	Grade 4	Grade 6		Grade 7	Grade 8	Grade 9	Grade 10	
			(int.)	(ext.)				(int.)	(ext.)
Age (0–1)	0.593	0.415	0.333	0.353	0.229	0.182	0.161	0.154	0.227
	(0.008)**	(0.008)**	(0.009)**	(0.010)**	(0.009)**	(0.010)**	(0.011)**	(0.012)**	(0.021)**
Female	0.172	0.218	0.293	0.135	0.347	0.337	0.341	0.333	0.076
	(0.005)**	(0.005)**	(0.005)**	(0.006)**	(0.007)**	(0.007)**	(0.008)**	(0.008)**	(0.012)**
Immigrant	− 0.218	− 0.226	− 0.231	− 0.411	− 0.251	− 0.231	− 0.250	− 0.332	− 0.735
	(0.012)**	(0.011)**	(0.011)**	(0.014)**	(0.014)**	(0.014)**	(0.013)**	(0.013)**	(0.025)**
Parents’ edu avg	0.337	0.354	0.345	0.436	0.327	0.309	0.261	0.196	0.402
	(0.007)**	(0.007)**	(0.007)**	(0.010)**	(0.008)**	(0.009)**	(0.008)**	(0.008)**	(0.016)**
Parents’ edu high	0.640	0.680	0.684	0.840	0.692	0.670	0.598	0.515	0.861
	(0.008)**	(0.008)**	(0.009)**	(0.012)**	(0.012)**	(0.012)**	(0.011)**	(0.012)**	(0.020)**
$R^2$	0.12	0.12	0.12	0.15	0.14	0.12	0.11	0.10	0.20
*N*	160,369	148,426	136,231	117,566	106,567	102,870	96,068	85,704	24,367

**$p<0$.01. Cohort FE included. Standard errors clustered by school. For each column, the sample used is the same than in Table 6

**Table 15:** Quantile regressions

	Grade 2	Grade 4	Grade 6		Grade 7	Grade 8	Grade 9	Grade 10	
			(int.)	(ext.)				(int.)	(ext.)
q15	0.480	0.321	0.230	0.421	0.136	0.119	0.100	0.078	0.206
	(0.012)**	(0.011)**	(0.013)**	(0.021)**	(0.014)**	(0.018)**	(0.022)**	(0.018)**	(0.036)**
q25	0.570	0.376	0.280	0.424	0.160	0.111	0.106	0.098	0.217
	(0.009)**	(0.012)**	(0.012)**	(0.016)**	(0.012)**	(0.016)**	(0.016)**	(0.013)**	(0.029)**
q35	0.612	0.429	0.334	0.406	0.178	0.132	0.105	0.083	0.179
	(0.008)**	(0.012)**	(0.012)**	(0.013)**	(0.011)**	(0.011)**	(0.013)**	(0.010)**	(0.028)**
q50	0.678	0.475	0.380	0.382	0.216	0.162	0.129	0.121	0.206
	(0.009)**	(0.011)**	(0.010)**	(0.010)**	(0.011)**	(0.009)**	(0.009)**	(0.008)**	(0.029)**
q65	0.692	0.489	0.415	0.343	0.261	0.196	0.161	0.141	0.200
	(0.010)**	(0.014)**	(0.011)**	(0.008)**	(0.013)**	(0.009)**	(0.013)**	(0.010)**	(0.022)**
q75	0.657	0.479	0.405	0.302	0.291	0.238	0.205	0.172	0.169
	(0.009)**	(0.015)**	(0.012)**	(0.009)**	(0.013)**	(0.012)**	(0.013)**	(0.014)**	(0.025)**
q85	0.579	0.422	0.376	0.262	0.303	0.261	0.231	0.206	0.148
	(0.010)**	(0.013)**	(0.012)**	(0.011)**	(0.015)**	(0.015)**	(0.015)**	(0.015)**	(0.022)**
*N*	160,438	148,472	136,281	117,752	106,707	102,960	96,170	86,025	32,053

**$p<0$.01. Regressors include dummies for gender, immigrant, parental education, and cohort. For each column, the sample used is the same than in Table 6

**Table 16:** Regressions with month or week dummies

	GPA grade 2		GPA grade 6		GPA grade 7		Repeat grade 1 or 2		Behind at 14		Enroll Academic	
	(M)	(W)	(M)	(W)	(M)	(W)	(M)	(W)	(M)	(W)	(M)	(W)
January/Week 1	0.519	0.569	0.299	0.329	0.180	0.132	− 0.593	− 0.688	− 0.356	− 0.351	0.144	0.050
	(0.011)**	(0.022)**	(0.012)**	(0.025)**	(0.014)**	(0.028)**	(0.034)**	(0.074)**	(0.016)**	(0.033)**	(0.037)**	(0.075)
November/Week 51	0.031	0.063	0.031	0.047	0.000	− 0.039	− 0.057	− 0.066	− 0.068	0.013	− 0.002	− 0.161
	(0.011)**	(0.021)**	(0.012)*	(0.024)+	(0.013)	(0.026)	(0.025)*	(0.053)	(0.016)**	(0.032)	(0.035)	(0.076)*
Female	0.170	0.170	0.293	0.293	0.339	0.339	− 0.121	− 0.121	− 0.283	− 0.283	0.416	0.416
	(0.005)**	(0.005)**	(0.005)**	(0.005)**	(0.007)**	(0.007)**	(0.013)**	(0.013)**	(0.007)**	(0.007)**	(0.016)**	(0.016)**
Immigrant	− 0.203	− 0.204	− 0.227	− 0.227	− 0.243	− 0.243	0.322	0.322	0.523	0.523	− 0.554	− 0.555
	(0.011)**	(0.011)**	(0.011)**	(0.011)**	(0.014)**	(0.014)**	(0.020)**	(0.020)**	(0.008)**	(0.008)**	(0.024)**	(0.024)**
Parents’ edu avg	0.326	0.326	0.342	0.341	0.323	0.323	− 0.364	− 0.365	− 0.396	− 0.397	0.503	0.503
	(0.006)**	(0.006)**	(0.007)**	(0.007)**	(0.008)**	(0.008)**	(0.016)**	(0.016)**	(0.007)**	(0.007)**	(0.017)**	(0.017)**
Parents’ edu high	0.621	0.621	0.679	0.679	0.680	0.680	− 0.705	− 0.705	− 0.899	− 0.899	1.110	1.112
	(0.008)**	(0.008)**	(0.009)**	(0.009)**	(0.012)**	(0.012)**	(0.024)**	(0.024)**	(0.011)**	(0.011)**	(0.025)**	(0.025)**
*N*	160,438	160,438	136,281	136,281	106,707	106,707	163,930	163,930	178,871	178,871	33,624	33,624

+$$p<0$$.1; *$$p<0$$.05; **$$p<0$$.01

**Table 17:** Regressions using students born around the cutoff date

	GPA 2	GPA 6	GPA 7	Repeat 1–2	Behind 14	Enroll Academic
Old	0.531	0.312	0.168	− 0.593	− 0.370	0.121
	(0.016)**	(0.017)**	(0.019)**	(0.049)**	(0.023)**	(0.050)*
				[− 0.043]	[− 0.108]	[0.041]
Female	0.172	0.305	0.327	− 0.157	− 0.273	0.422
	(0.016)**	(0.017)**	(0.020)**	(0.042)**	(0.023)**	(0.048)**
				[− 0.011]	[− 0.080]	[0.145]
Immigrant	− 0.181	− 0.257	− 0.302	0.326	0.497	− 0.581
	(0.027)**	(0.024)**	(0.028)**	(0.057)**	(0.028)**	(0.071)**
				[0.024]	[0.145]	[− 0.200]
Parents’ edu avg	0.344	0.343	0.323	− 0.387	− 0.393	0.453
	(0.019)**	(0.020)**	(0.022)**	(0.053)**	(0.026)**	(0.056)**
				[− 0.028]	[− 0.115]	[0.156]
Parents’ edu high	0.640	0.665	0.633	− 0.689	− 0.848	1.129
	(0.021)**	(0.024)**	(0.029)**	(0.071)**	(0.036)**	(0.076)**
				[− 0.050]	[− 0.247]	[0.388]
*N*	13,300	11,543	8856	13,572	14,978	2694

*$$p<0$$.05; **$$p<0$$.01

**Table 18:** Regressions with interaction terms (I)

	Retention		Evaluations					
	Repeat 1–2	Behind 14	2	4	6	6-ext.	7	8
Age at entry	− 0.671	− 0.381	0.543	0.406	0.349	0.383	0.207	0.148
	(0.043)**	(0.021)**	(0.017)**	(0.017)**	(0.017)**	(0.022)**	(0.019)**	(0.019)**
Age X parents’ edu avg	− 0.068	− 0.034	0.053	0.056	0.022	− 0.012	− 0.004	0.023
	(0.051)	(0.026)	(0.019)**	(0.019)**	(0.019)	(0.025)	(0.023)	(0.022)
Age X parents’ edu high	0.007	0.019	0.027	− 0.023	− 0.004	− 0.052	0.068	0.076
	(0.078)	(0.038)	(0.020)	(0.022)	(0.023)	(0.025)*	(0.025)**	(0.028)**
Age X female	0.085	0.018	0.028	0.015	0.009	0.013	0.044	0.051
	(0.048)+	(0.024)	(0.016)+	(0.016)	(0.017)	(0.020)	(0.020)*	(0.019)**
Age X immigrant	0.065	0.002	− 0.075	− 0.137	− 0.165	− 0.133	− 0.122	− 0.115
	(0.057)	(0.028)	(0.025)**	(0.023)**	(0.023)**	(0.030)**	(0.026)**	(0.025)**
Parents’ edu avg	− 0.337	− 0.380	0.300	0.321	0.331	0.441	0.326	0.294
	(0.027)**	(0.014)**	(0.011)**	(0.011)**	(0.012)**	(0.016)**	(0.014)**	(0.014)**
Parents’ edu high	− 0.707	− 0.907	0.608	0.682	0.681	0.863	0.647	0.623
	(0.038)**	(0.021)**	(0.013)**	(0.014)**	(0.014)**	(0.018)**	(0.017)**	(0.019)**
Female	− 0.158	− 0.291	0.156	0.211	0.288	0.128	0.317	0.313
	(0.023)**	(0.013)**	(0.009)**	(0.009)**	(0.010)**	(0.012)**	(0.012)**	(0.011)**
Immigrant	0.299	0.522	− 0.167	− 0.153	− 0.145	− 0.347	− 0.182	− 0.167
	(0.031)**	(0.016)**	(0.017)**	(0.015)**	(0.016)**	(0.021)**	(0.019)**	(0.018)**
*N*	163,910	178,871	160,438	148,472	136,281	117,752	106,707	102,960

+$$p<0.1$$; *$$p<0.05$$; **$$p<0.01$$

**Table 19:** Regressions with interaction terms (II)

	Graduate	Enroll		Special needs	
		Academic	Vocational	gr 1–2	gr 7–8
Age at entry	0.117	0.232	− 0.039	− 0.216	− 0.230
	(0.048)*	(0.047)**	(0.049)	(0.042)**	(0.043)**
Age X parents’ edu avg	0.113	0.138	− 0.146	− 0.120	0.043
	(0.058)+	(0.055)*	(0.060)*	(0.056)*	(0.060)
Age X parents’ edu high	− 0.055	− 0.037	− 0.092	0.136	0.111
	(0.077)	(0.074)	(0.080)	(0.066)*	(0.089)
Age X female	− 0.134	− 0.165	− 0.074	− 0.022	− 0.037
	(0.054)*	(0.051)**	(0.055)	(0.050)	(0.051)
Age X immigrant	− 0.074	− 0.178	0.143	− 0.100	0.012
	(0.060)	(0.066)**	(0.063)*	(0.070)	(0.060)
Parents’ edu avg	0.308	0.435	− 0.085	− 0.159	− 0.381
	(0.032)**	(0.033)**	(0.036)*	(0.034)**	(0.035)**
Parents’ edu high	0.774	1.128	− 0.501	− 0.455	− 0.641
	(0.050)**	(0.047)**	(0.047)**	(0.041)**	(0.051)**
Female	0.416	0.498	− 0.211	− 0.324	− 0.271
	(0.031)**	(0.031)**	(0.031)**	(0.027)**	(0.029)**
Immigrant	− 0.521	− 0.466	− 0.216	0.051	0.064
	(0.038)**	(0.039)**	(0.038)**	(0.041)	(0.037)+
*N*	33,624	33,624	33,624	149,142	115,204

+$$p<0.1$$; *$$p<0.05$$; **$$p<0.01$$

## Footnotes

1 Noticeable examples are the seminal work by Bedard and Dhuey (2006), where Spain is among the countries used to measure maturity effect on performance in TIMSS test in eight grade, and Sprietsma (2010), which uses results from a wave of PISA tests. However, the use of PISA tests for Spain can be problematic in the current setting, because they are administered in the spring of a given year to students who turn 16 that year. 16 years old is also the mandatory age of schooling in Spain, meaning that from then on students can leave education. Therefore, test takers born in January have already passed the mandatory age of schooling, while test takers born in December did not: the former group may not perfectly represent the total population of children born in January. See, for instance, PISA 2012 Technical Report, OECD, 2014.

2 To the best of our knowledge, no previous work focuses on Spain or one of its regions. Developed at the same time of this study, Berniell and Estrada (2017) use several waves of PISA tests to document that Spanish children born in December are more likely to have lower score and have repeated a grade than children born in January. They also use information from the Spanish population census to show that adults who were younger at school entry have less schooling. We compare some of our findings with theirs in Sect. 7.

3 E.g., Cook and Kang (2016) for USA In Catalonia, children get a degree at the end of tenth grade. As we discuss later in the text, incentives to stay in school and obtain the degree may be somehow stronger than in other countries, where a degree is awarded only at the end of the twelfth grade.

4 Decree 94/1992, issued on April, 28 (in Diari Official de la Generalitat de Catalunya (DOCG), núm. 1593-13/05/1992).

5 Decret 181/2008, issued on September, 9 (in DOGC núm. 5216-16/9/2008), and Order EDU/484/2009, issued on November 2, (in DOGC núm. 5505-13/11/2009).

6 In our data, about 97% of children enrolled for the first time in primary school in 2012 or 2013 were enrolled in preschool the year before. Some of the remaining 3% may have been enrolled in a semiprivate or private school. (Only enrollment data for public preschools are available to us.)

7 Statistics “Early leavers from education and training by sex and NUTS 2 regions” (edat_lfse_16) available on ec.europa.eu/eurostat.

8 For primary education: Decret 142/2007, issued on June, 26 (in DOGC núm. 4915-26/6/2007). For secondary education: Decret 143/2007, issued on June, 26 (in DOGC núm. 4915-26/6/2007).

9 Our data confirm that retention rules are enforced. For instance, among children born in 2003 (who normally enter primary education in 2009 and are in grade 5 by year 2013), only 30 (0.05%) were retained twice during the first two cycles of primary education. Among those born in 2001 (who typically complete the second and the third cycle of primary school before 2013), only 27 (0.04%) were retained twice in the time period spanned by our sample. Similarly for middle school, only 13 children born in 1997 and 22 children born in 1996 were retained three times from 2009 to 2013.

10 More than 30% of the students attend semiprivate schools (*Concertadas*), which are run privately and funded via both public and private sources. The remaining students attend a fully private school. For simplicity in this paper, we will define as “private” all schools that are not fully public.

11 Some schools initially report data only for their lower grades, covering the entire pool of students only after 2 or 3 years. Therefore, the sample used to study the outcomes in the lower grades is slightly larger.

12 We could not retrieve some or all the evaluations for about 2% of the students, who are excluded from the analysis in which evaluations are used as outcomes.

13 Statistics for students are similar in the previous years. However, as pointed out in Sect. 3.1, not all the schools joined the data collection at the same time. In 2009/2010, only 717 primary schools appear in the sample (496 middle schools) and their number sharply increases to 1440 in 2010/2011.

14 For simplicity, from now on we will refer to students whose nationality is not the Spanish one as “immigrant.” However, some of them may be second-generation immigrants; i.e., they may be born in Spain but not qualify for citizenship (e.g., if their parents just arrived in Spain).

15 The classification is made using only the level of information of one parent when information for the other parent is missing.

16 Special needs assignation is regulated by Llei Orgànica d’Educació LOE 2/2006 (articles 71 and 72).

17 In middle school, both numeric grades and the same five marks are assigned, and thus each word corresponds to an interval of numeric grades between 0 and 10. Using the same conversion scheme, we assign to each evaluation in primary school the midpoint of its interval; thus, “Fail” is interpreted as 3, “Pass” as 5, “Good” as 6, “Very good” as 7.5, and “Excellent” as 9.5. An alternative approach would be to just use numbers from 1 to 5. If the analyses discussed in the next section are replicated using this second approach, results are extremely similar.

18 For postponed enrollment, the share is slightly higher in 2012/2012, but the following year it drops back to 2011/2012 level. It is also worth noting that the statistics for 2009/2010 may be not fully comparable with the following years because the sample of schools involved is much smaller.

19 Several works of Cunha, Heckman and other co-authors study life cycle skill formation, showing that skill attainment at one stage of the life cycle raises skill attainment at later stages of the life cycle. Their finding supports the hypothesis that $\beta$ is positive and sizeable. See Cunha et al. (2006) for a review.

20 Performing the tests on older cohorts of students produces similar results.

21 Using a linear or higher order polynomial produces comparable results.

22 We use 8-year-old students to perform the tests to make sure that we are taking in account all children, also the few children who delayed the entrance. The *p* values of tests as the one in columns (1) and (2) of Table 4 are 0.3 and 0.6, respectively. For children of highly educated parents, they are 0.61 and 0.44, respectively.

23 Ideally, we would directly measure whether they have been retained at any point in time during the previous years, but we would need a longer panel to do so.

24 Results do not change if we restrict the sample in the third column to data used in the first column and the sample for the fifth column to the data used in the second column.

25 The age effect is significant and sizeable, although smaller in size, also used as dependent variable retention during second and third cycles of primary school. Even if children born in the last months of the year are overrepresented among those retained in the first cycle, it is still more likely for younger children to be retained throughout primary school.

26 As a robustness check, we restrict the estimation to students that we observe in two consecutive levels; for instance, for those born in 2003 or 2004, we have evaluations both in grade 2 and in grade 4. We can then perform the relevant regressions only on this subsample and compare the results with those obtained with the general specification. This simple robustness check always confirms the validity of our approach. Results are available upon requests.

27 While for the oldest cohorts, we can observe also students who are more than 1 year behind, this is not possible for 2005 cohorts. Given that the number of students who are two or more year behind or 1 year in advance is negligible, we ignore this difference. Further reducing the sample leaves the estimates unchanged.

28 When the final observation coincides with student’s last year in the sample, we do not have direct evidence that the student was promoted; however, given the choice of the sample, the regulation ensures that the student has to be promoted, except for very special cases. As a robustness check (not shown), we performed the same set of analysis exploiting one less cohort and imposing the further restriction that students are included in the sample only if we can observe them in a higher grade the following year. Results were completely equivalent to those discussed below.

29 In the interest of space, we do not report results for other grades. They are available upon requests.

30 See Calsamiglia and Loviglio (2018) for a more detailed discussion of children’s allocation to classes in primary and middle schools in Catalonia. We also replicate the analysis using peers at the school-year level, rather than at the class level, for both primary and secondary education, finding very similar results.

31 Coefficients are always significant but the magnitude of the effects is relatively small compared with the effect of own characteristics. In fact, variation in peers’ characteristics is relatively small within school. For instance, an increase of 0.1 in peers’ age at entry (which is much larger than one standard deviation) would decrease the GPA in second grade by 0.05 s.d., about one-third of the effect of increasing individual age by 3 months (i.e., about one standard deviation in expected age).

32 This result is in line with Arellano and Weidner (2016). Using as outcome the results in Trends in International Mathematics and Science Study (TIMSS) for a sample of Canadian students, they find that age effects are decreasing in ability.

33 However, it is noteworthy that the increasing and decreasing patterns, respectively, are very robust when performing censored quantile regressions that consider bottom or top outcomes as censored.

34 More precisely, we are identifying the effect of age at entry on the probability of concluding lower secondary education within the public system. We cannot follow students when they leave the public school and obtain a diploma through a private institution. However, we do observe enrollment in both public and private high school. We do not find graduation for 371 students that are then registered in the academic track (1% of the sample under analysis in this section). Given that middle school diploma is a compulsory requirement for enrollment in the academic high school, they should have obtained it in some unobservable way. We also observe 1722 students who enroll in vocational training without having completed lower secondary education in the public system. However, this fact does not provide sufficient evidence that they obtained a middle school diploma, because, after turning 17, students who previously drop out have the possibility to access vocational education after the successful completion of some preparatory courses (another event that we cannot observe in the data). Given that counting as graduated only the students enrolled in the academic track would slightly bias the results in our favor (older children are more likely to enroll there), we do not incorporate this information in our initial measure.

35 Results we obtained replicating the analyses described in this section for 15 years old are qualitatively similar, but higher in magnitude. The coefficient of the regressor might spuriously capture the fact that the lower tail of distribution of ability of older children is not in the sample; thus, older children in the sample had on average a better outcome. On the other hand, our estimates may be downward biased because of higher measurement error: some of the 14 years old that we count as dropout may be truly leaving our sample of interest at 15 because moving out of the Catalan education system.

36 We adopt this definition to allow the same delay for students in different grades at 14.

37 The share of the students with special needs related to learning is 2.8% in primary school and 3.2% in middle school. The corresponding figures for physical disabilities are 0.46% and 0.35%, respectively.

38 The only exceptions are May and August in grade 7, which are somewhat higher and somewhat lower, respectively. Given that highly educated parents are more likely to have children in May and less likely to have children in August, the dummies for these two months may be slightly biased by unobservable related to parental background.

39 In the interest of space, we do not show results for the other regressions. They are aligned with the results described in Sect. 5. Results are available upon requests.

40 Table 17 shows the estimated coefficients and marginal effects for a subset of outcomes: GPA (internal evaluations) in grades 2, 6, and 7, retention in the first two grades of primary school, being behind at 14 years old, enrollment in high school. Results for the remaining specifications are available upon request.

41 Our marginal effects are close enough to the estimates in Berniell and Estrada (2017). They use Spanish data from PISA tests and estimate a linear probability model to show that, among low SES, students who are born in December are 12.7 p.p. more likely to experience retention during compulsory education, while among high SES the gap decreases to 4 p.p. However, we think that this difference alone is not evidence of high SES investing more in their children if they are younger. In fact, as discussed, it can be observed also if high SES invest the same in children born in December and in children born in January, but they are on average more likely to have high-performing offspring.

42 There are no significant differences across the three categories of parental education when external evaluations in grade 10 are used. However, as explained in Sect. 5.2.1, students self-select into attending the last grade and therefore in taking the test. This can be a particularly relevant issue for the heterogeneity analysis because students with different parental backgrounds have different propensity to drop out. We do not report results for grades 9 and 10 in the table.

43 According to their results, the age effect on test scores is more than double for high SES than for low SES throughout primary education.

44 Younger children from high SES may foregone more inputs before the enrollment in preschool. However, those inputs may matter less for their performance in school.

45 Wald tests reject the null hypotheses that the effect is 0 for immigrants from grade 2 to grade 7. The null hypothesis cannot be rejected in grade 8.

46 Conditional on completion of lower secondary education, enrollment in high school is free and guaranteed to all applicants.

47 We test whether the sum of the coefficients of “Age” and “Age X female” is 0. The *p* values are 0.71 for graduation and 0.14 for enrollment in high school.

48
Loviglio (2019) finds that in Barcelona repeating a grade improves students’ cognitive skills but negatively affects their consumption value of schooling. The second effect is prevalent, and therefore, retained students are less likely to complete lower secondary education and enroll in academic upper secondary education.
